# Effects of adding quercetin to soybean meal-based feed on the growth, antioxidant capacity, and gut and liver health of juvenile turbot

**DOI:** 10.3389/fphys.2026.1950062

**Published:** 2026-10-28

**Authors:** Junfeng Hu, Lin Yang, Ziyu Jia, Shuyun Jiang, Xiaoqian Zhou, Yaxin Ge, Jianlong Du, Zhijie Dan

**Affiliations:** 1 Jiangsu Key Laboratory of Marine Bioresources and Environment, Jiangsu Ocean University, Lianyungang, China; 2 Makarov College of Marine Engineering, Jiangsu Ocean University, Lianyungang, China; 3 The Key Laboratory of Aquaculture Nutrition and Feed (Ministry of Agriculture and Rural Affairs), The Key Laboratory of Mariculture (Ministry of Education), Ocean University of China, Qingdao, China; 4 Jiangsu Institute of Marine Resources Development, Lianyungang, China

**Keywords:** antioxidant, gut and liver health, gut-liver axis, quercetin, *Scophthalmus maximus*

## Abstract

**Introduction:**

The antioxidant and anti-inflammatory properties of quercetin and their regulatory mechanisms had been extensively studied in aquatic animals. However, limited information was available regarding the effects of quercetin in farmed fish fed diets with high soybean meal inclusion. The purpose of this study was to investigate the effects of adding quercetin to soybean meal-based feed on the growth, antioxidant capacity, and gut and liver health of juvenile turbot.

**Methods:**

Five isonitrogenous and isolipidic experimental diets were formulated: a fish meal diet (FM), a diet in which 40% of the fish meal was replaced with soybean meal (SBM), and three diets based on the SBM diet, each supplemented with quercetin at graduated levels of 0.05%, 0.1%, and 0.2%, designated as Q0.05, Q0.1, and Q0.2, respectively. These diets were randomly assigned to 15 tanks (200 L), with 30 juvenile turbot (initial weight 8.41 ± 0.52 g) reared in each tank for a feeding trial of 70 days.

**Results:**

The results showed that, compared with the FM group, the specific growth rates (SGR) in the SBM group decreased significantly (*P* < 0.05). As dietary quercetin levels increased, SGR exhibited an increasing trend, although no significant differences were observed among groups. Among the antioxidant indicators, there was no significant difference in MDA levels between the FM and SBM groups. After the addition of quercetin, MDA levels in the quercetin-supplemented groups decreased significantly, and the Q0.1 group had the lowest MDA levels (*P* < 0.05). Simultaneously, the Q0.1 group had the highest total antioxidant capacity (T-AOC) levels. Among the gut morphological indicators, the FM group and the quercetin-supplemented groups both showed significantly higher villus height, muscular layer thickness, and goblet cell count than the SBM group (*P* < 0.05). Furthermore, the Q0.2 group had the highest villus height and goblet cell count (*P* < 0.05), and the Q0.05 group had the highest muscular layer thickness (*P* < 0.05). Among the indicators for gut inflammatory genes and tight junction protein genes, the results for both the FM group and the quercetin-supplemented groups were significantly better than those of the SBM group (*P* < 0.05). *claudin-7* and *occludin* mRNA expression in the Q0.05 group were significantly upregulated compared to the SBM group (*P* < 0.05). Among the gut microbiota indicators, the FM group had significantly higher Shannon index and Simpson index than the SBM group (*P* < 0.05), and the Q0.1 group had the highest Shannon index and Simpson index compared to the SBM group (*P* < 0.05). At the same time, the gut microbiota composition in the Q0.1 group showed significant improvement. Serum level of lipopolysaccharide (LPS) in the FM and quercetin supplement groups were significantly lower than those in the SBM group (*P* < 0.05), and the LPS in the Q0.1 group was significantly lower than those in the SBM group (*P* < 0.05). Serum level of diamine oxidase (DAO) in the Q0.1 was significantly lower than those in the SBM group (*P* < 0.05). Alanine aminotransferase (ALT) activity and aspartate aminotransferase (AST) activity in the FM group were significantly lower than those in the SBM group (*P* < 0.05), the ALT activity in the Q0.2 group were significantly lower than those in the SBM group (*P* < 0.05), and AST activity in the Q0.05 group were significantly lower than those in the SBM group (*P* < 0.05). In the Q0.05 group, both γ-glutamyltransferase (γ-GT) activity and triglycerides (TG) content were significantly lower than those in the SBM group (*P* < 0.05). Liver sections revealed that, compared with the FM group, the SBM group exhibited partial vacuolization of hepatocytes, swelling and rupture of the endoplasmic reticulum, widespread lipid deposition, and elevated levels of reactive oxygen species (ROS). After adding quercetin, the above conditions improved effectively. Furthermore, compared with the SBM group, lipid deposition was significantly reduced in the Q0.1 group, and ROS levels was significantly reduced in the Q0.05 group (*P* < 0.05). Regarding the expression of inflammatory genes in the liver, expression levels in the SBM group were generally higher than those in the FM and the quercetin-supplemented group. The *tnf-α* and *stat3* mRNA expression levels in the Q0.1 group were significantly lower than in the SBM group (*P* < 0.05).

**Discussion:**

In summary, dietary quercetin supplementation in diets with 40% fish meal replacement by soybean meal significantly enhanced antioxidant capacity and protected gut and liver health in turbot, with concomitant improvements in growth performance. The gut–liver axis was possibly involved in this regulatory process. The optimal dietary quercetin supplementation level was 0.2% in the present study.

## 1 Introduction

Seafood is key to achieving food and nutrition security goals (Willett et al., 2019). In recent years, declining marine fishery stocks and the swift expansion of intensive aquaculture cause an imbalance in fishmeal supply and demand (Hayes, 2023). Resulting in persistently high prices (Tran et al., 2022). For aquaculture industry to achieve continuous and sustainable production growth, alternative plant protein materials must be explored to replace conventional fishmeal. In terms of feed protein supply, soybean meal is a superior plant-derived protein material with competitive pricing and balanced nutrition (Li et al., 2025). However, studies have shown that high levels of soybean meal in feed can lead to problems such as liver and gut inflammation. Consequently, the use of green feed additives to mitigate these issues has become a hot topic in current research.

The “gut-liver axis” refers to the close connection established between the gut and the liver through a bidirectional network of interactions at the anatomical, physiological, and molecular levels, facilitating the exchange of gut and liver signals generated by dietary, genetic, and environmental factors (Judith et al., 2020). LPS and DAO are important markers in research on the gut-liver axis. When intestinal mucosal cells are damaged and intestinal mucosal permeability increases, large amounts of LPS and DAO released from within the cells pass through the intestinal mucosal barrier into the bloodstream, resulting in a decrease in LPS and DAO activity within the intestine. Therefore, LPS and DAO are important markers of intestinal barrier damage (Ghosh et al., 2020; Wen et al., 2014). Studies have shown that upregulating the gut-liver axis can mitigate the damage caused by a high-carbohydrate diet in grass carp (Wang et al., 2025). The findings of Gao et al. (2026) indicated that chitosan can activate the MAPK/NF-κB signaling pathway via the gut-liver axis, thereby enhancing the growth and immune response of black bass in recirculating aquaculture systems. In addition, a study by Luan et al. (2026) on zebrafish also demonstrated that microcystins can cause liver toxicity in zebrafish, whereas the probiotic *Lactobacillus rhamnosus* can modulate the gut microbiota. *Lactobacillus rhamnosus* first improves disturbances in gut lipid metabolism and organic acid metabolism, and then alleviates inflammatory response in the liver and oxidative stress via the gut-liver axis, thereby effectively regulating liver health. To date, limited research has been conducted regarding gut-liver axis responses to dietary soybean meal replacement. Therefore, this study investigated the effects of quercetin on the growth, antioxidant capacity, and gut-liver health of juvenile turbot by adding quercetin to soybean meal-based feed.

Quercetin is a polyhydroxyflavonoid with the molecular formula C_15_H_10_O_7_ (Kandemir et al., 2022). Fruits and vegetables contain rich quantities of this substance, including apples, berries and tea, as well as in many traditional Chinese medicinal herbs, such as acacia seeds, acacia flowers, moutan bark, plantain seeds, and ginkgo leaves (Anand et al., 2016). Quercetin is an important flavonoid compound that has been extensively studied as a novel feed additive. In mammals, quercetin has been shown to diminish oxidative stress status in mice (Kobori et al., 2015) and alleviate *E. coli*-induced liver damage in broiler chickens (Zhou et al., 2025), and also effectively increased egg and milk production and regulated gut oxidative status and inflammation (Abd El-Aziz et al., 2025). In the field of aquaculture, quercetin can enhance disease resistance capacity and stress endurance of farmed fish (Li et al., 2019), and boost the growth indicators and immune defense capacity of aquatic species (Porras et al., 2017). Furthermore, in research exploring dietary fishmeal replacement by soybean meal, the addition of quercetin not only enhanced the antioxidant capacity of spotted black bass but also significantly reduced the transmittance of their gut mucosa, improved the immunity and structural integrity of gut tissues, increased the relative abundance of Proteobacteria, and to some extent, elevated the abundance and diversity of gut microflora (Liu et al., 2024a), highlighting its potential as a green and effective alternative to antibiotic supplementation (Doñate et al., 2010).

The turbot (*Scophthalmus maximus* L.), commonly known as “ Duobao Fish “ is a carnivorous species characterized by rapid growth, delicate and palatable flesh, and high nutritional value (Ma et al., 2021). It is an economically important species in China’s marine aquaculture industry (Liu et al., 2015). However, high levels of soybean meal in the diet can lead to liver and gut inflammation in turbot (Dan et al., 2022; Wang et al., 2023a). Therefore, this study focused on turbot and investigated the influences brought by supplementation with distinct concentrations of quercetin to soybean meal-based feed to determine how quercetin mitigated the adverse effects of high soybean meal replacement on turbot via the gut-liver axis. This study could supply theoretical references and practical guidance for the application of quercetin as a natural antioxidant feed additive to mitigate high soybean meal stress in juvenile turbot farming. Meanwhile, it could lay a research foundation for exploiting plant polyphenols to optimize high soybean meal diets within marine fish aquaculture.

## 2 Materials and methods

### 2.1 Experimental design and diet preparation

Quercetin was purchased from Shanxi Nanba Biotechnology Co., Ltd, purity≥95%. Five isonitrogenous and isolipidic experimental diets were formulated: a fish meal diet (FM), a diet in which 40% of the fish meal was replaced with soybean meal (SBM), and three diets based on the SBM diet, each supplemented with quercetin at graduated levels of 0.05%, 0.1%, and 0.2%, designated as Q0.05, Q0.1, and Q0.2, respectively. The quercetin dosage was based on the studies by Hoseini et al. (2025) and Liu et al. (2024b). These diets were randomly assigned to 15 tanks (200 L), with 30 juvenile turbot (initial weight 8.41 ± 0.52 g) reared in each tank for a feeding trial of 70 days. First, various solid ingredients were ground using a grinder. A stepwise titration approach was adopted to mix trace elements, with fish oil and soy lecithin added subsequently, which were mixed thoroughly and sieved through an 80-mesh screen. Water, choline chloride, and dimethyl-β-propiothetin (DMPT) were then added and mixed thoroughly. Then processed into strip-shaped feed with a cross-sectional diameter of 2 mm using a strip cutter, dried in an oven at 65°C, cut into pellets approximately 5 mm in length, packaged in resealable bags with labels, and stored at -20°C for future use (**Table 1**).

**Table 1:** Composition and nutrient levels of diets (dry matter, %).

Items	Groups				
	FM	SBM	Q0.05	Q0.1	Q0.2
Ingredients					
Fish meal ^1^	55.00	33.00	33.00	33.00	33.00
Soybean meal ^1^	0.00	31.50	31.50	31.50	31.50
Wheat gluten meal ^1^	9.45	10.00	10.00	10.00	10.00
Wheat meal ^1^	20.00	11.50	11.50	11.50	11.50
α-Starch ^1^	4.45	0.41	0.36	0.31	0.21
Fish oil	3.50	5.20	5.20	5.20	5.20
Soybean lecithin	2.00	2.00	2.00	2.00	2.00
L-Methionine	0.00	0.32	0.32	0.32	0.32
Lysine	0.00	0.30	0.30	0.30	0.30
Histidine	0.00	0.17	0.17	0.17	0.17
Sodium alginate	1.00	1.00	1.00	1.00	1.00
Vitamin C phosphate ester	0.05	0.05	0.05	0.05	0.05
Vitamin and mineral premix ^2^	1.50	1.50	1.50	1.50	1.50
Yttrium trioxide	0.10	0.10	0.10	0.10	0.10
Choline chloride	0.30	0.30	0.30	0.30	0.30
Antiseptic ^3^	0.10	0.10	0.10	0.10	0.10
Ethoxyquin	0.05	0.05	0.05	0.05	0.05
Attractants ^4^	0.50	0.50	0.50	0.50	0.50
Squid visceral meal	1.00	1.00	1.00	1.00	1.00
Taurine	0.50	0.50	0.50	0.50	0.50
Ca (H_2_PO_4_)_2_	0.50	0.50	0.50	0.50	0.50
Quercetin	0.00	0.00	0.05	0.10	0.20
Total	100.00	100.00	100.00	100.00	100.00
Nutrient levels					
Moisture	1.94	2.44	2.16	1.86	1.67
Crude ash	10.93	9.68	9.62	9.64	9.66
Crude lipid	10.20	9.93	9.66	10.19	10.40
Crude protein	51.54	51.84	51.37	50.79	50.45

^1^ Fishmeal (93.19 % dry matter, 72.89 % crude protein, 8.04 % crude lipid); Soybean meal (90.36 % dry matter, 50.91 % crude protein, 0.35 % crude lipid); wheat gluten meal (91.25 % dry matter, 70.50 % crude protein, 0.64 % crude lipid); Wheat meal (86.81 % dry matter, 13.70 % crude protein, 0.45 % crude lipid); α-Starch (95.43 % dry matter, 0.20 % crude protein, 0.10 % crude lipid). All of the feed ingredients were provided by Shandong Haiding Farming Co. Ltd. (Jinan, China).

^2^ Vitamin and mineral premix was supported by Qingdao Master Biotechnology Co., Ltd.

^3^ Antiseptic: calcium propionate: boletic acid = 1:1.

^4^ Attractants: glycine betaine: dimethyl-β-propiothetin: glycine: alanine: inosine-5-diphosphate trisodium salt = 4: 2: 2: 1: 1.

### 2.2 Laboratory fish and rearing management

Juvenile turbot used in this study were purchased from Huanghai Aquatic Products Co., Ltd., Haiyang City, Shandong Province. To facilitate acclimation to the rearing conditions, the fish were fed commercial feed for 14 consecutive days. After acclimation, the fish were randomly assigned to five dietary treatments with three replicate tanks per group (200 L; 30 fish per tank). Fish were fed to apparent satiation twice daily at 07:00 and 19:00 throughout the feeding trial of 70 days. A flow-through water system was adopted for rearing. Continuous aeration was supplied by air pumps, and the water temperature was maintained at 18-22°C during the experimental period.

### 2.3 Sample collection

After the feeding trial concluded, the fish were fasted for 24 hours, and the turbot in each tank were weighed and counted. From every tank, 23 turbots were picked at random before rapid eugenol anesthesia, with the initial six fish measured for body length and weighed. Disposable medical syringes were used to obtain blood from the caudal vein of turbot. Blood samples were centrifuged at 3,000 rpm for 10 minutes at 4°C to prepare serum, which was then stored in a -80°C ultra-low temperature freezer for future used. Once blood sampling was completed, dissection was performed on the fish to reveal the abdominal cavity, the visceral mass was removed and weighed, and a soybean-sized piece of liver was extracted. One portion was frozen in dry ice for molecular enzyme activity assays and ROS sections, and another portion is placed in 4% polyformaldehyde and electron microscopy fixative for hematoxylin-eosin (H&E) sections, Oil Red O sections, and electron microscopy sections. Additionally, a soybean-sized section of the gut was taken, one portion is frozen in dry ice for molecular enzyme activity assays, and the other portion is placed in 4% polyformaldehyde for H&E and AB-PAS sections. Finally, a sample of the hindgut mucosa was collected for gut microbiota analysis.

All experimental procedures involving animals were reviewed and approved by the Animal Care and Use Committee of Jiangsu Ocean University.

### 2.4 Growth performance indicators

Specific growth rate (SGR, %/day) = 100 × [ln (final weight) -ln (initial weight)]/days

Weight gain rate (WGR, %) = 100× (final weight - initial weight)/initial weight

Feed efficiency (FE) = (final weight - initial weight)/Dry feed intake

Feed conversion ratio (FCR) = Dry feed intake/Weight gain

Viscerosomatic index (VSI, %) = 100 ×Viscera weight/Body weight

Hepatosomatic index (HSI, %) = 100× Liver weight/Body weight

Survival rate (SR, %) = 100 × (final number of fish/initial number of fish)

Condition factor (CF, g · cm^−3^) = 100× Body weight/(Total length)^3^

### 2.5 Antioxidant parameters

Serum activities of serum superoxide dismutase (SOD), catalase (CAT), glutathione (GSH), total antioxidant capacity (T-AOC) and content of malondialdehyde (MDA) were measured using commercial assay kits purchased from Nanjing Jiancheng Bioengineering Institute. Detailed detection procedures were provided in the **Supplementary Materials**.

### 2.6 Gut digestibility indices

Serum content of total protein (TP), and activities of trypsin (TRY), α-amylase (AMS) and lipase (LPS) were measured using commercial assay kits purchased from Nanjing Jiancheng Bioengineering Institute. Detailed detection procedures were provided in the **Supplementary Materials**.

### 2.7 Morphological structure of liver and gut tissues

For the preparation of hepatic and intestinal H&E sections, liver and gut tissues isolated from juvenile turbot were first trimmed to a suitable size, rinsed with running tap water, dehydrated through an ethanol gradient, cleared in xylene, and embedded in paraffin. A paraffin microtome was employed to prepare tissue sections at a thickness of 4-5 μm. After air drying, the sections were routinely dewaxed to water, followed by H&E staining, hydrochloric acid-alcohol differentiation, bluing with running tap water, graded ethanol dehydration, xylene clearing, and mounting with neutral resin. Observation of tissue microstructure was conducted via light microscopy in the final step, with digital images collected for further analysis. Image J software was used to measure the villus height, villus width, and muscle layer thickness in each group.

Preparation of OCT-embedded liver sections was described below: Livers isolated from juvenile turbot were fixed, embedded in OCT compound, and cryosectioned in accordance with the experimental standard operating procedures (SOPs) to obtain tissue sections. After fixation and membrane permeabilization treatment, the sections were subjected to staining, differentiation, nuclear counterstaining and mounting procedures. Afterwards, the cryosections were stained and mounted. Finally, light microscopy was applied to observe tissue morphological features, and digital photos were recorded to support further analysis.

The procedure for liver transmission electron microscopy (TEM) sample preparation was described as follows: Liver tissues were harvested from juvenile turbot, rapidly dissected and trimmed to approximately 1 mm³ within 1–3 min, and immediately transferred into TEM fixative for fixation and storage at 4°C. Samples were first rinsed using 0.1 mol/L phosphate-buffered saline (pH 7.4), then fixed with 1% osmium tetroxide at room temperature away from light over a 2-hour period. The samples were subsequently dehydrated with an ethanol–acetone gradient, followed by permeabilization and embedding in embedding resin. Resin blocks were fabricated via polymerization at 60°C for 48 h. The resin blocks were sectioned into 1.5 μm semi-thin sections, which were localized by toluidine blue staining, thereafter, 60–80 nm ultrathin sections were cut using an ultramicrotome. A double staining procedure was performed using uranyl acetate and lead citrate as staining agents, and transmission electron microscopy was used to observe the sections and capture micrographs for subsequent analysis.

The procedure for preparing intestinal AB-PAS sections was described as follows: Firstly, the sections were dewaxed with an environmentally friendly dewaxing solution, followed by rehydration through an ethanol gradient. For frozen sections, tissues were thawed prior to fixation and washing. Subsequently, the sections were stained with AB-PAS Solution C, acidified with Solution B, and stained with Solution A under light-shielded conditions. Lastly, the sections were dehydrated via an ethanol gradient and cleared with xylene, after which embedding in neutral resin was conducted. Finally, the samples were observed under a microscope, and ImageJ software was utilized to process captured micrographs and quantify goblet cell numbers.

The procedure for preparing liver ROS sections was described as follows: The frozen sections were thawed at room temperature and blotted dry, and regions of interest were marked with a histochemical pen. The ROS detection probe was diluted with PBS at a ratio of 1:500 to prepare the ROS staining working solution. The diluted ROS working solution was applied to the tissue, and the sections were protected from light and incubated in a constant-temperature and humidity chamber at 37°C for 30 min. After that, three successive 5-min washes were performed by shaking the slides in pH 7.4 phosphate-buffered saline on a decolorization rack. DAPI stain was added dropwise, and the slides were incubated away from light at ambient temperature for 10 min. Subsequently, three separate 5-min washing cycles were conducted on a shaking decolorization rack after the slides were soaked in pH 7.4 PBS. An anti-fade mounting medium was applied to cover the specimen slides. Finally, images were acquired.

### 2.8 Real-time quantitative PCR testing

All samples were crushed into powder under liquid nitrogen and stored for subsequent analyses. The powder was placed in a centrifuge tube containing Trizol and steel beads and fully ground. After centrifugation, the supernatant was collected. Chloroform was added, and the mixture was thoroughly mixed and left for layered separation. The aqueous phase was collected, and isopropanol was added for RNA precipitation. Following centrifugation, the supernatant was discarded, and the precipitate was washed with 75% ethanol. Residual liquid was removed after another round of centrifugation, and the dried RNA pellet was dissolved in DEPC-treated water. The purity of the extracted RNA was assessed by measuring the 260/280 absorbance ratio. cDNA was synthesized from the isolated RNA using HiScript II Reverse Transcriptase. The forward and reverse primer sequences of each target gene were synthesized by GenScript Biotech (Shanghai) Co., Ltd., with β-actin used as the internal reference gene (**Table 2**).

**Table 2:** Sequences of primers for quantitative real-time PCR.

Primers	Forward primer (5′- 3′)	Reverse primer (5′- 3′)	Genbank no.
*β-actin*	CGTGCGTGACATCAAGGAG	AGGAAGGAAGGCTGGAAGAG	EU686692.1
*tnf-α* ^1^	GGGTGGATGTGGAAGGTGAT	GGCCTCTGTTTGGCTTGACT	XM_035638223.1
*ifn-γ* ^1^	GCTTTCCCGATCATCTTCTG	GGTTTCCCAGATTCCCATTC	DQ400686.1
*tlr2* ^1^	GCACTTACTCTGGATCTGTCCTTCAA	TACGTGGTGGAGCTCATGAGTTT	AMQ35497
*stat2* ^1^	TGTCGGACCTAAATAAGCTCCT	CTCTGCCACTCCACTAGCTC	XM_035630581.2
*stat3* ^1^	CCAACATCCTGGTTTCACCTC	CTTCAATGGGGAAGGCCGAT	NW_024040041.1
*stat5a* ^1^	GCTCCTCAGAGGGTGTTGAAG	GTGGGTTCTCCAGGTCTATGG	NW_024040041.1
*tricellulin*	GCCTACATCCACAAAGACAACG	TCATTCCCAGCACTAATACAATCAC	KU238183
*claudin-7*	CATTTCAGCAAACACATC	CGGCACAGAAATCCCAGAG	XM_038715821.1
*occludin*	ACTTTGGATTACCTGCG	GGGAGGGGCAAGACAACAGT	XM_038734217.1
*p38* ^1^	GAACGCCCCCAACATCTCTA	CTCGGCTGCTGTTATTCGC	SRP172919
*nrf-2* ^1^	GGGTGGATGTGGAAGGTGAT	GGCCTCTGTTTGGCTTGACT	AWP13156.1
*jnk* ^1^	CTGGTAGAGCAGGTAGGACA	CACAGAAGACACTGGAAGAA	XM_035644965
*bcl-2* ^1^	TTATCAGCGGCATCTTCATCTC	TTGGCGAGGCGGTGTAATC	XM_020104180.1
*mtor* ^1^	ACCGATCTGCCGTCATCATT	TGGAGCGGAAAAAGCCTTGA	XM_035644862

^1^ *tnf-α*, tumornecrosisfactor-α; *ifn-γ*, interferon-γ; *tlr2*,Toll-like receptor 2; *stat2*, *stat3*, *stat5a*, signal transducer and activator of transcription-2, 3, 5a; *p38*, tumor suppressor gene38; *nrf2*, nuclear factor erythroid-2 related factor; *jnk*, c-Jun N-terminal kinase; *bcl-2*, B-cell lymphoma-2; *mtor*, mechanistic target of rapamycin.

Assays were performed using a quantitative PCR kit on the PQD-96a V1.0.13 RC 20220921 for research platform. The 20 μL reaction mixture consisted of 10 μL SYBR qPCR Master Mix, 0.4 μL each of forward and reverse primers, 2 μL cDNA, and 7.2 μL ddH_2_O. Primer specificity was verified using melting curve analysis. PCR amplification program: 95°C for 30 seconds, 95°C for 10 seconds, 60°C for 30 seconds, repeat for 40 cycles. Relative expression levels were calculated using the 2^-ΔΔCt^ method.

### 2.9 Gut microbiota

Firstly, total genomic DNA was extracted using the cetyltrimethylammonium bromide/sodium dodecyl sulfate method. The 16S rRNA gene was amplified with forward and reverse primers. The amplified region covered the V4 region of 16S rRNA, the sequence of the forward primer was GTGCCAGCMGCCGCGGTAA, and the sequence of the reverse primer was GGACTACHVGGGTWTCTAAT. Each PCR mixture was supplemented with 15 µL of Phusion^®^ High-Fidelity PCR Master Mix (New England Biolabs), 0.2 µM of each primer, and 10 ng of genomic DNA template. The mixture was denatured at 98°C for 1 min, followed by 30 cycles consisting of 98°C for 10 s, 50°C for 30 s, and 72°C for 30 s. A final extension was conducted at 72°C for 5 min. The PCR products were examined via electrophoresis on a 2% agarose gel. Qualified PCR products were purified with magnetic beads, quantified by enzyme-linked immunosorbent assay, and pooled at equal volumes according to their concentrations. After thorough mixing, the pooled PCR products were separated on a 2% agarose gel by electrophoresis, and target bands were recovered. Subsequently, sequencing libraries were constructed. The constructed libraries were quantified using Qubit and qPCR. After quality verification, the libraries were sequenced on the NovaSeq6000 platform with the PE250 strategy. Raw sequencing data were assigned to individual samples according to Barcode sequences and PCR primer sequences. Valid data were acquired through paired-end read assembly, quality filtering, and chimera removal. Noise reduction was performed using the DADA2 module embedded in QIIME2 (Version QIIME2-202202), and representative ASVs were obtained. Rapid multiple sequence alignment was implemented in QIIME2 to resolve phylogenetic relationships among all ASV sequences. Finally, the sequencing data of each sample were normalized, Alpha diversity, Beta diversity and LEfSe analyses were carried out, and Venn diagrams and species abundance bar charts were generated accordingly.

### 2.10 Gut barrier integrity

Serum contents of diamine oxidase (DAO) and lipopolysaccharide (LPS) were performed in accordance with the instructions provided with the ELISA kit from Jiangsu Jingmei Biotechnology Co., Ltd.

A one‐step sandwich ELISA method based on two antibodies was adopted by the ELISA kit. Standard wells and sample wells were arranged on the microplate. A volume of 50 μL standards with different concentrations was added to each standard well, and 10 μL of test specimens supplemented with 40 μL of sample diluent was sequentially loaded into each sample well. A total of 100 μL of horseradish peroxidase (HRP)‐conjugated secondary antibody was added to all standard and sample wells. The microplate was sealed with sealing film and incubated at 37°C for 60 min. The waste liquid was aspirated completely, and the residual liquid in the wells was dried with absorbent filter paper. The wells were then fully filled with washing buffer. After static incubation for 1 min, the washing solution was discarded by shaking, and the microplate was blotted dry again. The above washing procedure was repeated five times. Equal volumes (50 μL) of Substrate A and Substrate B were added to each well, and the microplate was incubated at 37°C in the dark for 15 min. Subsequently, 50 μL of stop solution was added to each well to terminate the reaction, and the absorbance values at 450 nm were measured within 15 min. Finally, a standard curve was constructed, and the corresponding formula was established for the calculation of sample concentrations.

### 2.11 Serum physio-biochemical indices

Serum contents of triglycerides (TG), albumin (ALB), and serum activities of alanine aminotransferase (ALT), aspartate aminotransferase (AST), alkaline phosphatase (AKP) and gamma-glutamyltransferase (γ-GT) were measured using commercial assay kits purchased from Nanjing Jiancheng Bioengineering Institute. Detailed detection procedures were provided in the **Supplementary Materials**.

### 2.12 Statistical analysis

Statistical analyses were performed using SPSS Version 26.0. Firstly, the data were examined for homogeneity. One-way ANOVA was conducted followed by Tukey’s *post hoc* multiple comparisons when no significant differences in homogeneity were detected. The difference was considered statistically significant when *P* < 0.05. Data were expressed as means ± standard error (S.E.).

## 3 Results

### 3.1 The growth performance

There was no significant difference in SGR values among the quercetin-supplementation groups (*P* > 0.05), and the SGR in the FM group was significantly higher than that in the SBM group (*P* < 0.05). There was no significant difference in WGR among the SBM and the quercetin-supplementation groups (*P* > 0.05), and the WGR in the FM group was significantly higher than that in the SBM group (*P* < 0.05). There were no significant differences in FE among the FM, SBM, Q0.05, and Q0.2 groups (*P* > 0.05). However, the FE for the Q0.1 group was lower than that of the SBM group (*P* < 0.05). There was no significant difference in FCR among the FM, SBM, Q0.05, and Q0.2 groups (*P* > 0.05). However, the FCR in the Q0.1 group was higher than that in the SBM group (*P* < 0.05). No significant intergroup differences in VSI existed for FM, SBM, Q0.05 and Q0.2 groups (*P* > 0.05), but the VSI in the Q0.1 group was significantly lower than that in the SBM group (*P* < 0.05). There were no significant differences in HSI among the FM, SBM, and Q0.2 groups (*P* > 0.05). However, the HSI in the SBM group was significantly higher than that in the Q0.05 and Q0.1 groups (*P* < 0.05). There were no significant differences in SR and CF among the different treatment groups (*P* > 0.05) (**Table 3**).

**Table 3:** Effects of quercetin supplementation in high-soybean-meal diets on growth performance and survival of juvenile turbot. ^1^.

Parameters	FM	SBM	Q0.05	Q0.1	Q0.2	*p*-Value
SGR (%/day) ^2^	2.66 ± 0.06^a^	2.27 ± 0.08^b^	2.4 ± 0.12^ab^	2.29 ± 0.01^ab^	2.4 ± 0.03^ab^	0.028
WGR (%) ^2^	543.69 ± 26.54^a^	365.83 ± 19.31^b^	438.5 ± 46.87^ab^	396.61 ± 3.00^b^	436.91 ± 9.63^ab^	0.017
FE ^2^	1.21 ± 0.02^a^	1.17 ± 0.01^a^	1.20 ± 0.05^a^	0.91 ± 0.04^b^	1.18 ± 0.19^a^	0.000
FCR ^2^	0.83 ± 0.01^b^	0.85 ± 0.01^b^	0.90 ± 0.11^b^	1.13 ± 0.05^a^	0.86 ± 0.15^b^	0.000
VSI (%) ^2^	5.98 ± 0.42^ab^	7.45 ± 0.68^a^	5.67 ± 0.03^ab^	5.28 ± 0.06^b^	5.63 ± 0.08^ab^	0.044
HSI (%) ^2^	1.27 ± 0.14^ab^	1.45 ± 0.02^a^	0.9 ± 0.10^b^	1.01 ± 0.00^b^	1.01 ± 0.01^ab^	0.019
SR (%) ^2^	100 ± 0.00	100 ± 0.00	100 ± 0.00	98.89 ± 1.92	100 ± 0.00	0.452
CF (g·cm^-3^) ^2^	3.52 ± 0.03	3.23 ± 0.14	3.29 ± 0.01	3.45 ± 0.07	3.72 ± 0.20	0.140

^1^ Data were presented as mean ± SD (n=3). Means in the same row sharing the same superscript letter are not significantly different determined by Tukey's test (*P >*0.05).

^2^ SGR, Specific growth rate; WGR, Weight gain rate; FE, Feed efficiency; FCR, Feed conversion ratio; VSI, Viscerosomatic index; HSI, Hepatosomatic index; SR, Survival rate; CF, Condition factor.

### 3.2 Antioxidant capacity

No significant difference in serum MDA content was observed among the FM, SBM, and Q0.05 groups (*P* > 0.05). However, the MDA content in the SBM group was significantly higher than that in the Q0.1 and Q0.2 groups (*P* < 0.05). There were no significant differences in serum T-AOC levels among the FM, SBM, Q0.05, and Q0.2 groups (*P* > 0.05). However, the T-AOC level in the Q0.1 group was significantly higher than that in the FM group (*P* < 0.05). SOD, CAT and GSH showed no significant differences among the different treatment groups (*P* > 0.05) (**Table 4**).

**Table 4:** Effects of quercetin supplementation in high-soybean meal diets on antioxidant capacity of juvenile turbot. ^1^.

Indices	FM	SBM	Q0.05	Q0.1	Q0.2	*p*-Value
MDA (nmol/ml) ^2^	9.53 ± 1.53^ab^	14.51 ± 2.24^a^	6.82 ± 0.89^ab^	4.47 ± 0.47^b^	5.35 ± 0.53^b^	0.011
SOD (U/ml) ^2^	156.63 ± 14.49	144.52 ± 12.31	144.92 ± 1.99	134.99 ± 1.19	144.32 ± 2.58	0.578
CAT (U/ml) ^2^	0.45 ± 0.05	0.97 ± 0.47	2.29 ± 0.78	1.14 ± 0.19	1.85 ± 0.07	0.127
GSH (U/mgprot) ^2^	1293.94 ± 89.05	1136.67 ± 171.82	893.33 ± 24.85	1154.55 ± 38.79	1011.21 ± 182.12	0.236
T-AOC (mmol/gprot) ^2^	0.80 ± 0.05^b^	0.93 ± 0.13^ab^	1.14 ± 0.20^ab^	1.51 ± 0.13^a^	1.39 ± 0.03^ab^	0.035

^1^ Data were presented as mean ± SD (n=3). Means in the same row sharing the same superscript letter are not significantly different determined by Tukey's test (*P >*0.05).

^2^ MDA, Malondialdehyde; SOD, Superoxide dismutase; CAT, Catalase; GSH, Glutathione; T-AOC, Total antioxidant capacity.

### 3.3 Gut digestibility indicators

No significant statistical divergence was observed for lipase activity across the quercetin-supplementation groups (*P* > 0.05), and the lipase activity in the SBM group was significantly lower than that in the FM group (*P* < 0.05). There were no significant differences in AMS and TRY among the different treatment groups (*P* > 0.05) (**Table 5**).

**Table 5:** Effects of quercetin supplementation in high‐soybean meal diets on gut digestibility of juvenile turbot. ^1^.

Indices	FM	SBM	Q0.05	Q0.1	Q0.2	*p*-Value
AMS (U/mgprot) ^2^	0.21 ± 0.02	0.16 ± 0.03	0.13 ± 0.01	0.19 ± 0.01	0.17 ± 0.02	0.084
Lipase (U /gprot) ^2^	20.89 ± 3.4^a^	9.45 ± 0.61^b^	13.06 ± 1.26^ab^	16.07 ± 0.82^ab^	16.88 ± 1.48^ab^	0.012
TRY ( U/mgprot) ^2^	2226.08 ± 364.3	2054.72 ± 205.11	2413.7 ± 930.2	1988.65 ± 881.95	2901.44 ± 285.72	0.833

^1^ Data were presented as mean ± SD (n=3). Means in the same row sharing the same superscript letter are not significantly different determined by Tukey's test (*P >*0.05).

^2^ AMS, α-amylase; TRY, Trypsin.

### 3.4 Morphology of gut tissue

The gut mucosal tissue structure of the FM group was intact and well-defined, with tightly and orderly arranged villi, in contrast, the SBM group exhibited reduced villus height and a less dense and intact structure. After adding quercetin to the high-soybean-meal diet, gut villus height increased, and the arrangement became denser (**Figure 1**). No significant differences in villus height existed when comparing SBM and Q0.05 groups (*P* > 0.05). However, villus height in the FM, Q0.1, and Q0.2 groups was significantly higher than that in the SBM group (*P* < 0.05). Microvillus height did not differ significantly among SBM and Q0.1 groups (*P* > 0.05). However, microvillus height in the FM, Q0.05, and Q0.2 groups was significantly higher than that in the SBM group (*P* < 0.05). There was no significant difference in muscle layer thickness among the FM and Q0.1 groups (*P* > 0.05). Muscle layer thickness in the FM, Q0.05, and Q0.2 groups was significantly higher than that in the SBM group (*P* < 0.05). No significant difference in goblet cell numbers were detected when comparing FM and quercetin- supplementation groups (*P* > 0.05), and the number of goblet cells in the SBM group was significantly lower than that in the FM and quercetin-supplementation groups (*P* < 0.05) (**Table 6**).

**Figure 1:**
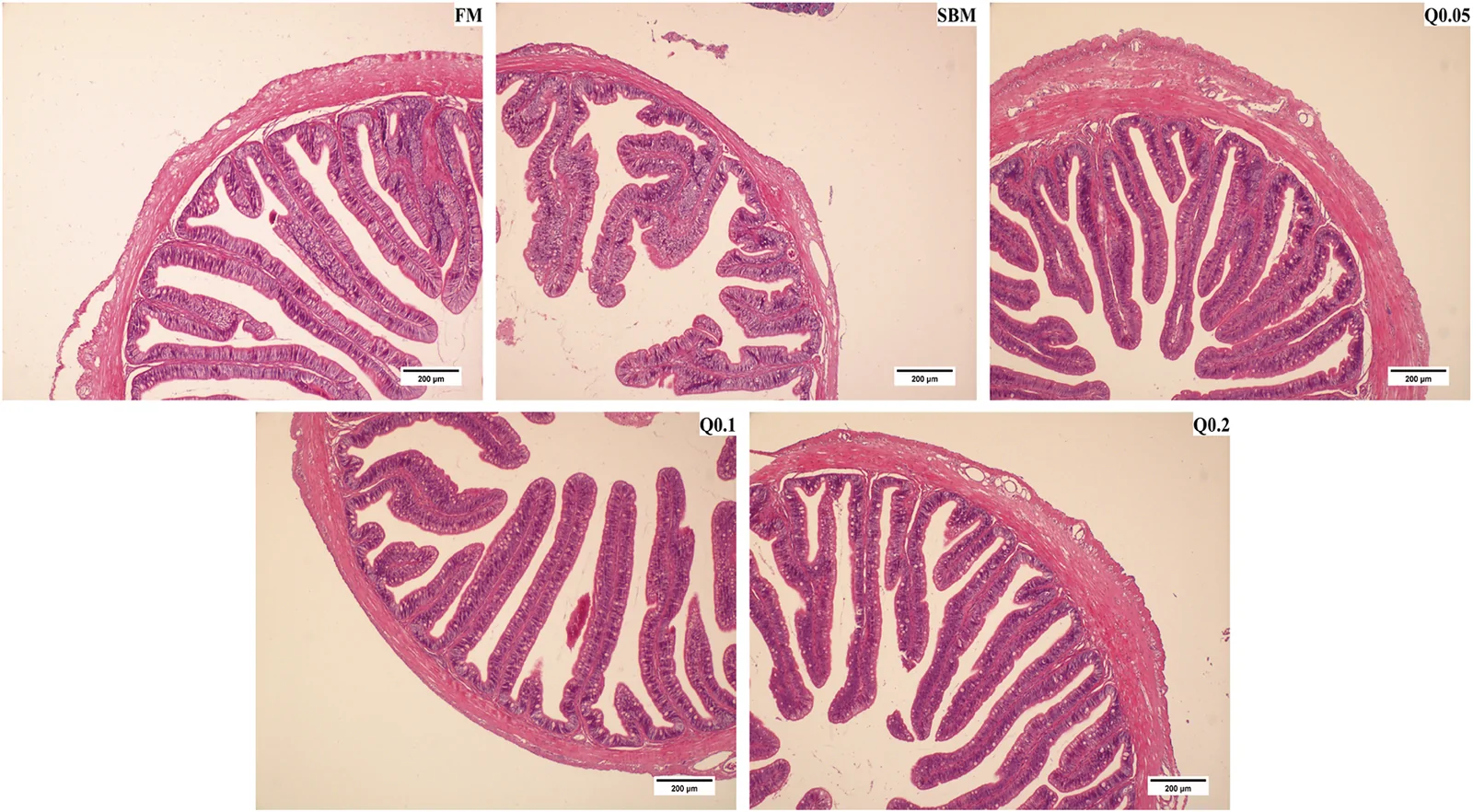
Effects of quercetin supplementation in high-soybean meal diets on gut histomorphology of juvenile turbot. Images of HE-stained sections (100 ×, scale bar: 200 μm) (n=3).

**Table 6:** Effects of quercetin supplementation in high-soybean meal diets on gut morphological structure of juvenile turbot. ^1^.

Indices	FM	SBM	Q0.05	Q0.1	Q0.2	*p*-Value
Villus height (μm)	963.52 ± 38.92^a^	540.93 ± 78.18^b^	743.89 ± 21.27^ab^	861.72 ± 31.63^a^	865.32 ± 49.13^a^	0.001
Microvillus height (μm)	11.05 ± 0.57^a^	3.76 ± 0.66^b^	9.09 ± 0.54^a^	6.02 ± 0.43^b^	10.34 ± 0.75^a^	0.000
Muscular thickness (μm)	136.35 ± 2.05^b^	35.81 ± 3.95^c^	212.88 ± 4.66^a^	132.93 ± 3.99^b^	197.75 ± 2.04^a^	0.000
Goblet cell (cells/HPF)	9769.00 ± 910.31^ab^	3692.67 ± 101.86^c^	7496.33 ± 791.06^b^	7443.67 ± 462.19^b^	11600.67 ± 826.29^a^	0.000

^1^ Data were presented as mean ± SD (n=3). Means in the same row sharing the same superscript letter are not significantly different determined by Tukey's test (*P >*0.05).

### 3.5 Expression of gut-associated genes

There were no significant differences in *mtor* mRNA expression levels among the FM, SBM, Q0.05, and Q0.1 groups (*P* > 0.05). *mtor* mRNA expression levels in the Q0.2 group were significantly higher than those in the FM, SBM, Q0.05, and Q0.1 groups (*P* < 0.05). There were no significant differences in *bcl-2* mRNA expression levels among the FM, SBM, Q0.05, and Q0.1 groups (*P* > 0.05). *bcl-2* mRNA expression in the Q0.2 group was significantly higher than in the SBM and Q0.1 groups (*P* < 0.05). Comparisons among SBM and quercetin-supplementation groups revealed no significant difference in *nrf-2* mRNA expression (*P* > 0.05), and *nrf-2* mRNA expression in the FM group was significantly higher than that in the SBM and quercetin-supplementation groups (*P* < 0.05). Comparisons of FM and quercetin-supplementation groups displayed no significant difference in *p38* mRNA expression (*P* > 0.05), and *p38* mRNA expression levels in the SBM group were significantly higher than those in the FM and the quercetin-supplementation groups (*P* < 0.05). There were no significant differences in *jnk* mRNA expression levels among the different treatment groups (*P* > 0.05) (**Figure 2**).

**Figure 2:**
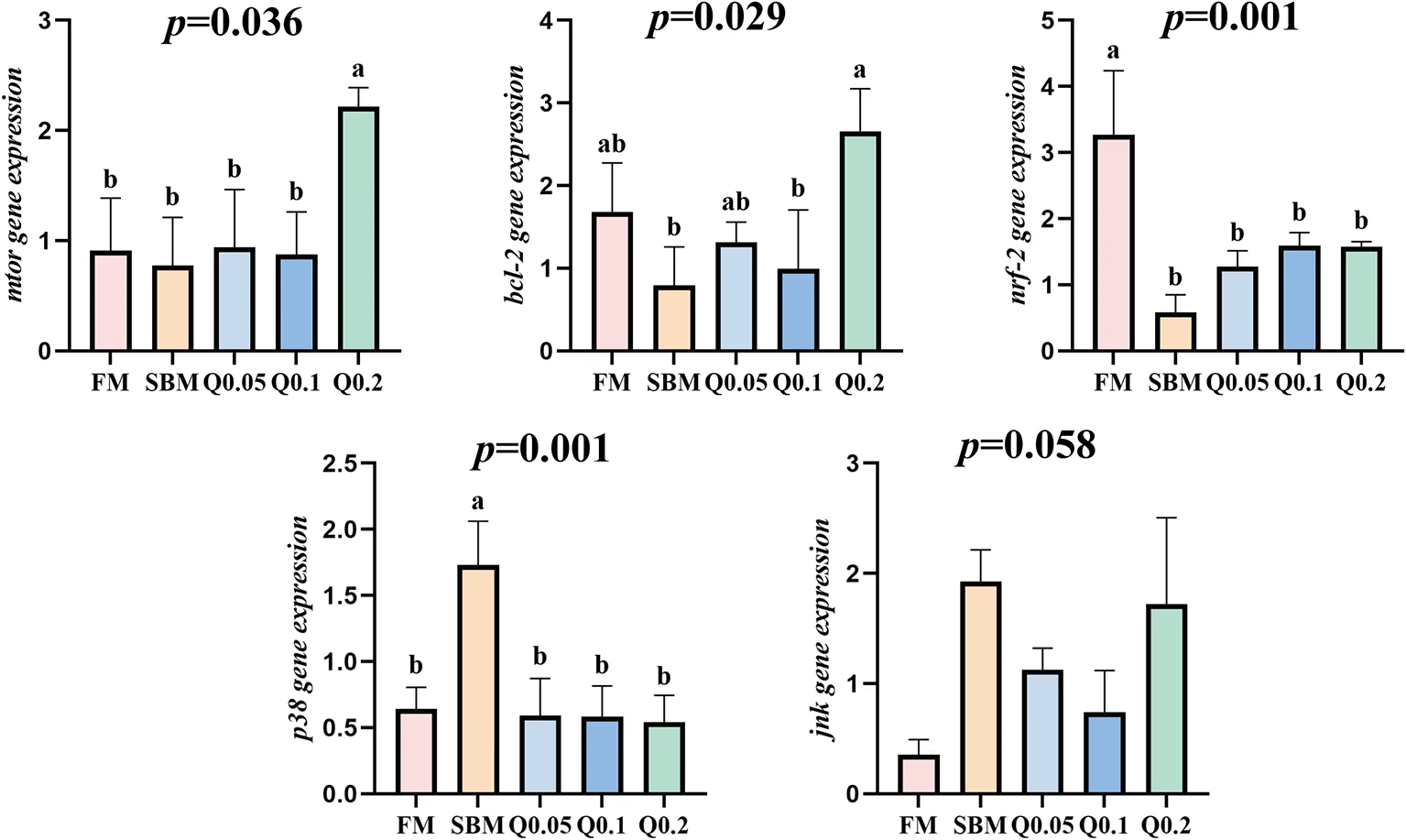
Effects of quercetin supplementation in high-soybean meal diets on gut inflammatory gene expression of juvenile turbot. Data were expressed as mean ± SEM and analyzed by One-way ANOVA, followed by Tukey’s multiple range test (n=3). Means in the same row sharing the same superscript letter are not significantly different determined by Tukey's test (*P >*0.05).

There were no significant differences in *claudin-7* mRNA expression levels among the FM, SBM, Q0.1, and Q0.2 groups (*P* > 0.05). *claudin-7* mRNA expression levels in the FM and SBM groups were significantly lower than those in the Q0.05 group (*P* < 0.05). There were no significant differences in *occludin* mRNA expression levels among the FM, SBM, Q0.1, and Q0.2 groups (*P* > 0.05), and *occludin* mRNA expression in the SBM group was significantly lower than that in the Q0.05 group (*P* < 0.05). No significant difference in *tricellulin* mRNA expression were found among the different treatment groups (*P* > 0.05) (**Figure 3**).

**Figure 3:**
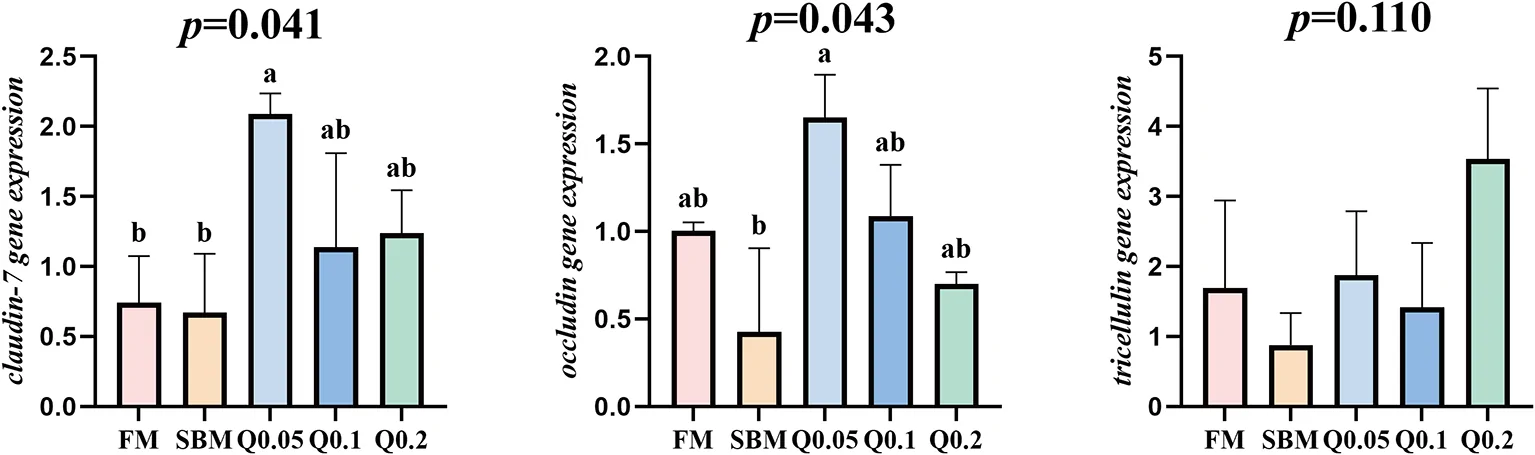
Effects of quercetin supplementation in high-soybean meal diets on gut tight junction protein gene expression of juvenile turbot. Data were expressed as mean ± SEM and analyzed by One-way ANOVA, followed by Tukey’s multiple range test (n=3). Means in the same row sharing the same superscript letter are not significantly different determined by Tukey's test (*P >*0.05).

### 3.6 Gut microbiota

#### 3.6.1 Gut microbiome diversity

There was no significant difference in the Shannon index among the SBM, Q0.05, and Q0.2 groups (*P* > 0.05), and the Shannon index in the SBM group was significantly lower than that in the FM and Q0.1 groups (*P* < 0.05). There was no significant difference in the Simpson index among the quercetin-supplementation groups (*P* > 0.05), and the Simpson index in the SBM group was significantly lower than that in the FM group (*P* < 0.05). Furthermore, the Shannon index and Simpson’s index for the Q0.1 group were significantly higher than those for the SBM group (*P* < 0.05). There was no significant difference in the treatment groups among coverage and the Chao-1 index (*P* > 0.05) (**Table 7**).

**Table 7:** Effects of dietary quercetin supplementation on gut microbiota alpha diversity of juvenile turbot fed high-soybean meal diets. ^1^.

Indices	FM	SBM	Q0.05	Q0.1	Q0.2	*p*-Value
Good’s coverage (%)	100.00 ± 0.00	100.00 ± 0.00	100.00 ± 0.00	100.00 ± 0.00	100.00 ± 0.00	–
Chao1 index	528.98 ± 112.3	173.64 ± 44.31	231.61 ± 1.83	516.87 ± 186.99	238.99 ± 7.42	0.076
Shannon index	6.97 ± 0.43^a^	1.38 ± 0.22^c^	2.84 ± 0.05^bc^	3.44 ± 0.59^b^	2.76 ± 0.06^bc^	0.000
Simpson index	0.97 ± 0.01^a^	0.32 ± 0.11^c^	0.66 ± 0^b^	0.69 ± 0.04^b^	0.64 ± 0.01^b^	0.000

^1^ Data were presented as mean ± SD (n=3). Means in the same row sharing the same superscript letter are not significantly different determined by Tukey's test (*P >*0.05).

The number of shared OTUs across the five groups was 59. Compared with the FM group, the SBM group had a higher number of unique OTUs, though a slight distinction could be seen, no statistical significance was attained. Within the quercetin-supplementation groups, the Q0.1 group had the highest number of unique OTUs (1,092), and the Q0.05 group had the lowest (320) (**Figure 4A**).

**Figure 4:**
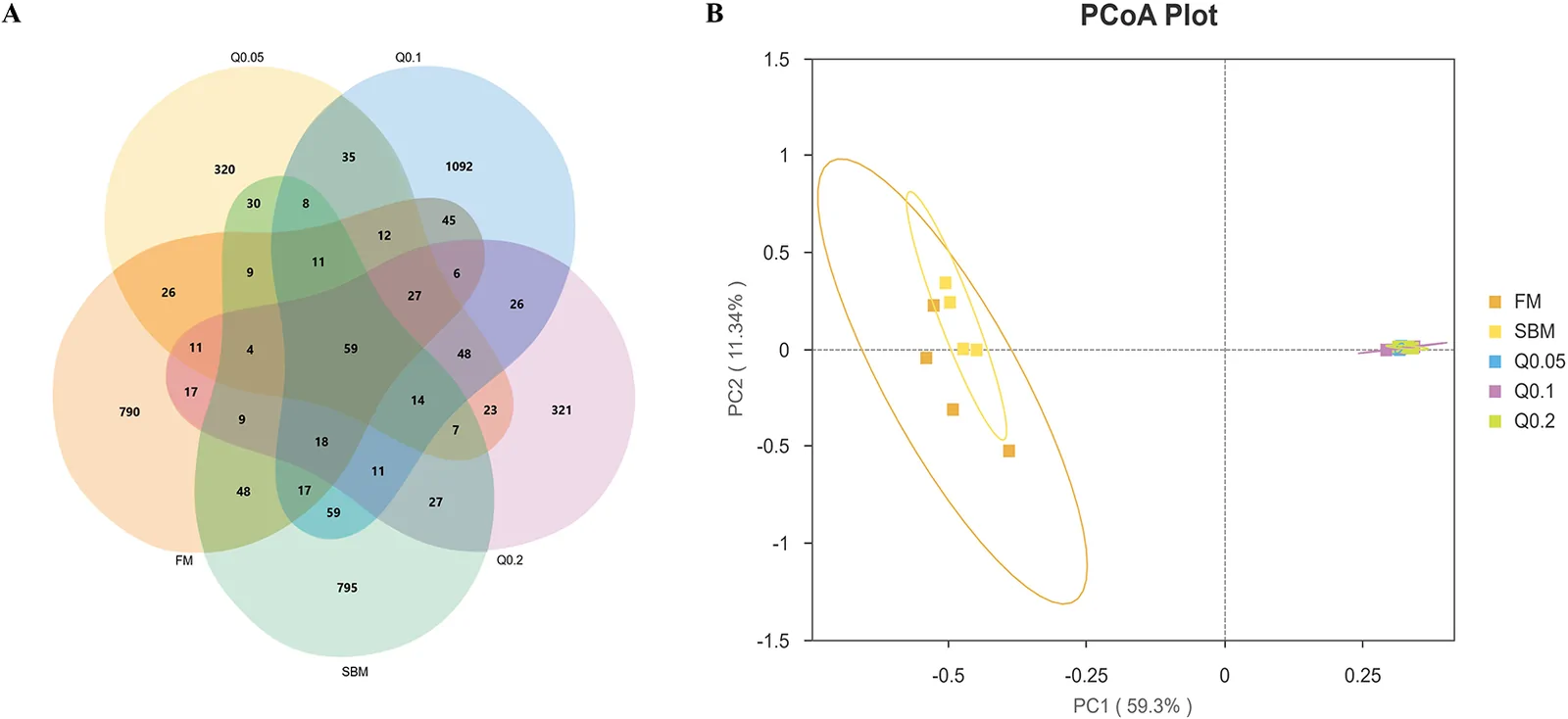
Effects of dietary quercetin supplementation on gut microbial community structure of juvenile turbot fed high-soybean meal diets (n=3). **(A)** Venn diagram showed the number of shared and unique OTUs among the groups; **(B)** PCoA showed the β-diversity of the gut microbiota composition among the groups.

Statistical analysis confirmed remarkable intergroup divergence in the gut flora profiles of juvenile turbot. The PC1 fully distinguished samples from the FM and SBM groups, indicating that the high-soybean-meal diet significantly altered the gut microbiota composition, whereas the treatment groups supplemented with quercetin in the high-soybean-meal diet exhibited distinct clustering and were clearly separated from the SBM group. Furthermore, the microbial profiles of these three groups showed high clustering, suggesting that quercetin can significantly improve the high-soybean-meal-induced disruption of the gut microbiota structure, and that there were no significant differences in the regulatory effects among the different supplementation levels (**Figure 4B**).

#### 3.6.2 Composition of the gut microbiota

At the phylum level, the gut microbiota of juvenile turbot primarily consisted of the Pseudomonadota, Bacillota, Bacteroidota and Halobacteriota. Among these, the relative abundances of the Pseudomonadota in the five groups were 34.84%, 47.72%, 95.37%, 90.71%, and 95.68%, respectively. At the genus level, the gut microbiota of juvenile turbot in the FM group mainly consisted of *Photobacterium* (0.95%), *Vibrio* (1.92%), *Streptococcus* (3.46%), and *Acinetobacter* (1.75%), and that of juvenile turbot in the SBM group mainly consisted of *Photobacterium* (22.28%), *Vibrio* (14.52%), and *Halococcus* (2.73%), and the gut microbiota of juvenile turbot in the quercetin-supplementation groups primarily consisted of *Aeromonas*, *Pseudomonas*, and *Stenotrophomonas*. The relative abundances of *Aeromonas* were 57.81%, 55.73%, and 58.98%, respectively, those of *Pseudomonas* were 18.9%, 17.88%, and 18.64%, respectively, and the relative abundances of the genus *Stenotrophomonas* were 9.37%, 8.72%, and 9.59%, respectively (**Figure 5**).

**Figure 5:**
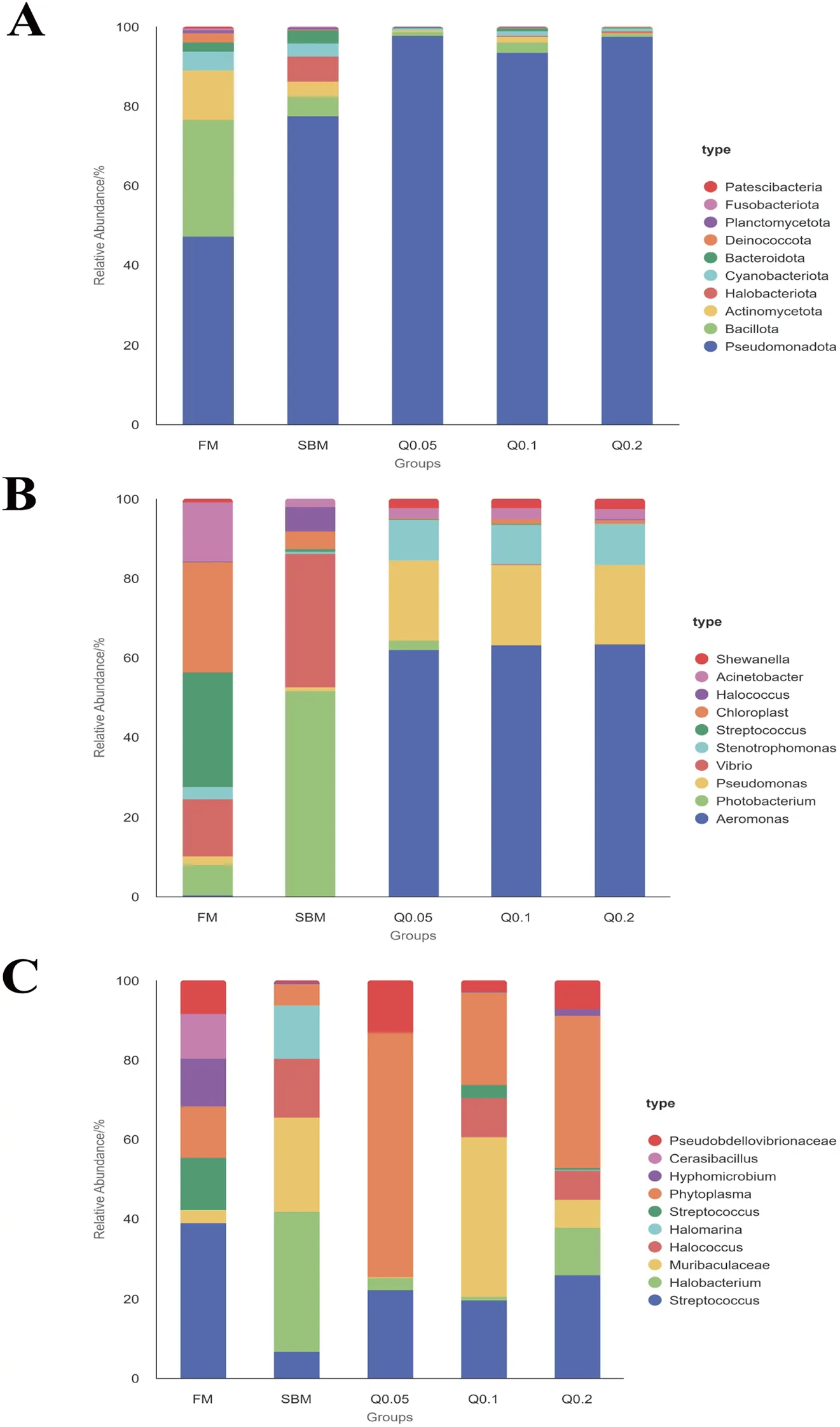
Effects of quercetin supplementation in high-soybean meal diets on the relative abundance of gut microbiota of juvenile scophthalmus maximus at phylum, genus and species levels (n=3). **(A)** Phylum level. **(B)** Genus level. **(C)** Species level. The abscissa was the sample name and the ordinate was the relative abundance percentage. Different colors represented different species.

There were significant differences in the characteristics of the gut microbiota between the FM and SBM groups of juvenile turbot (LDA score > 4). The characteristic microbiota of the FM group consisted primarily of the *Actinobacteriota* phyla, with the *Bacilli* class, *Streptococcaceae* family, *Lactococcus* genus, and *Micrococcaceae* family been the main enriched taxa, exhibiting high LDA values. In contrast, the number of characteristic microbial communities in the SBM group was significantly reduced, and the group was dominated by halophilic archaea and Proteobacteria, including the genera *Halococcus*, the family *Vibrionaceae*, and the order *Enterobacteriales*. Divergent differential species compositions among groups illustrated significant structural shifts in the intestinal flora of juvenile turbot fed high-soybean-meal feeds. In the quercetin-supplementation groups, the core biomarkers of the Q0.05 group were *Photobacterium*, *Fimbriiglobus*, and *Pseudobdellovibrionaceae*, which were mainly symbiotic functional bacteria typical of fish. The Q0.1 group was enriched with a large number of environmental bacterial communities, including the *Chloroflexota*, *Acidobacteriota* and *Lachnospiraceae*. Group Q0.2 is characterized primarily by environmental *Proteobacteria*, such as the genus *Psychrobacter* and the order *Caulobacterales* (**Figure 6**).

**Figure 6:**
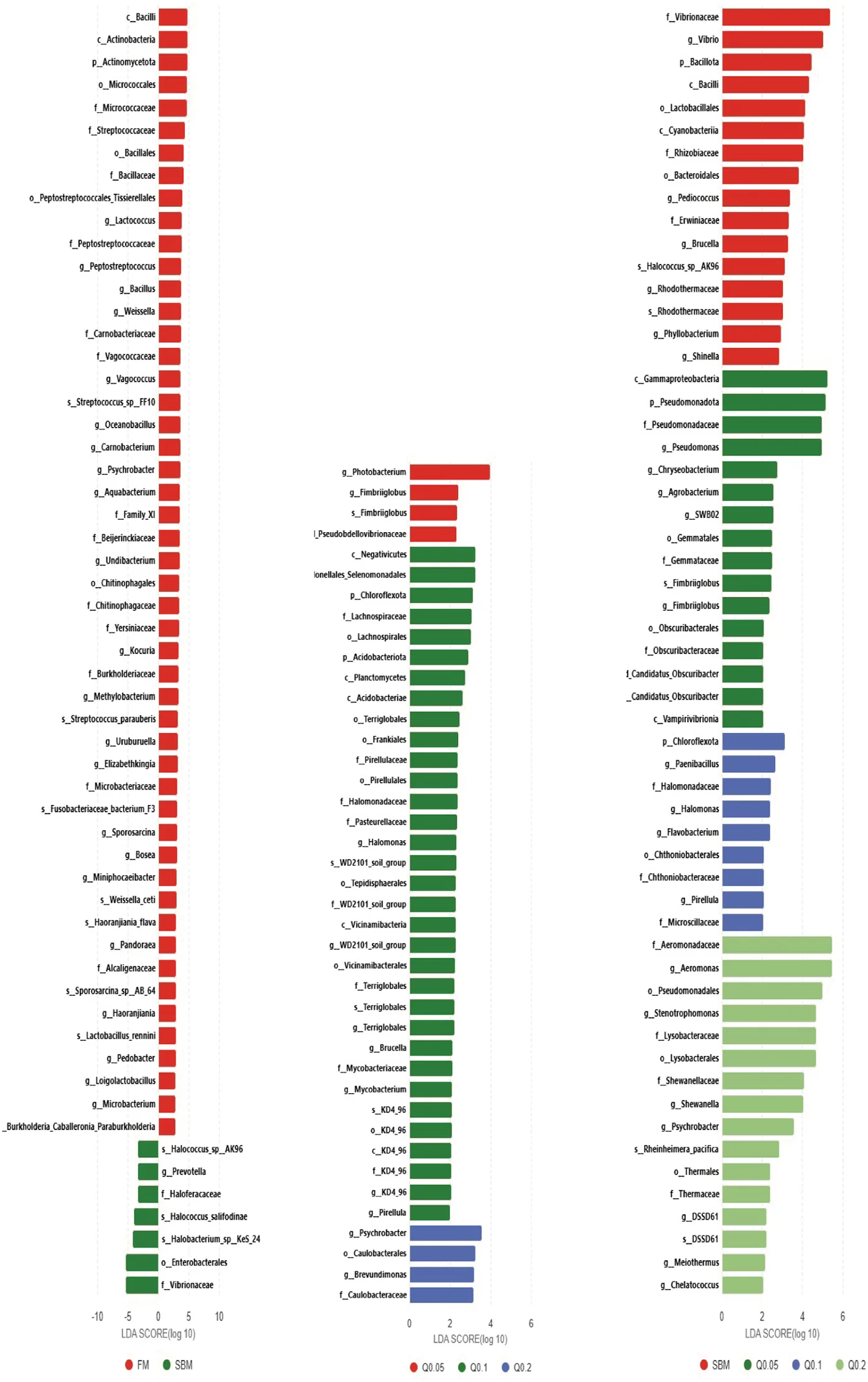
LEfSe analysis of differential gut microbiota in juvenile fed high-soybean meal diet supplemented with quercetin (n=3). LEfSe identified the most differentially abundant taxon among different groups. Significantly discriminant taxon nodes were colored and branch areas were shaded according to the effect size of taxa. Taxa enriched in the group were indicated with LDA scores. Only taxa meeting an LDA significant threshold of 2 were shown.

### 3.7 Gut barrier integrity indicators

There was no significant difference in LPS content among the FM and the quercetin-supplementation groups (*P* > 0.05), and the LPS content in the SBM group was significantly higher than that in the Q0.1 and Q0.2 groups (*P* < 0.05). There were no significant differences in DAO content among the FM, SBM, Q0.05, and Q0.2 groups (*P* > 0.05). However, the DAO content in the SBM group was significantly higher than that in the Q0.1 group (*P* < 0.05) (**Figure 7**).

**Figure 7:**
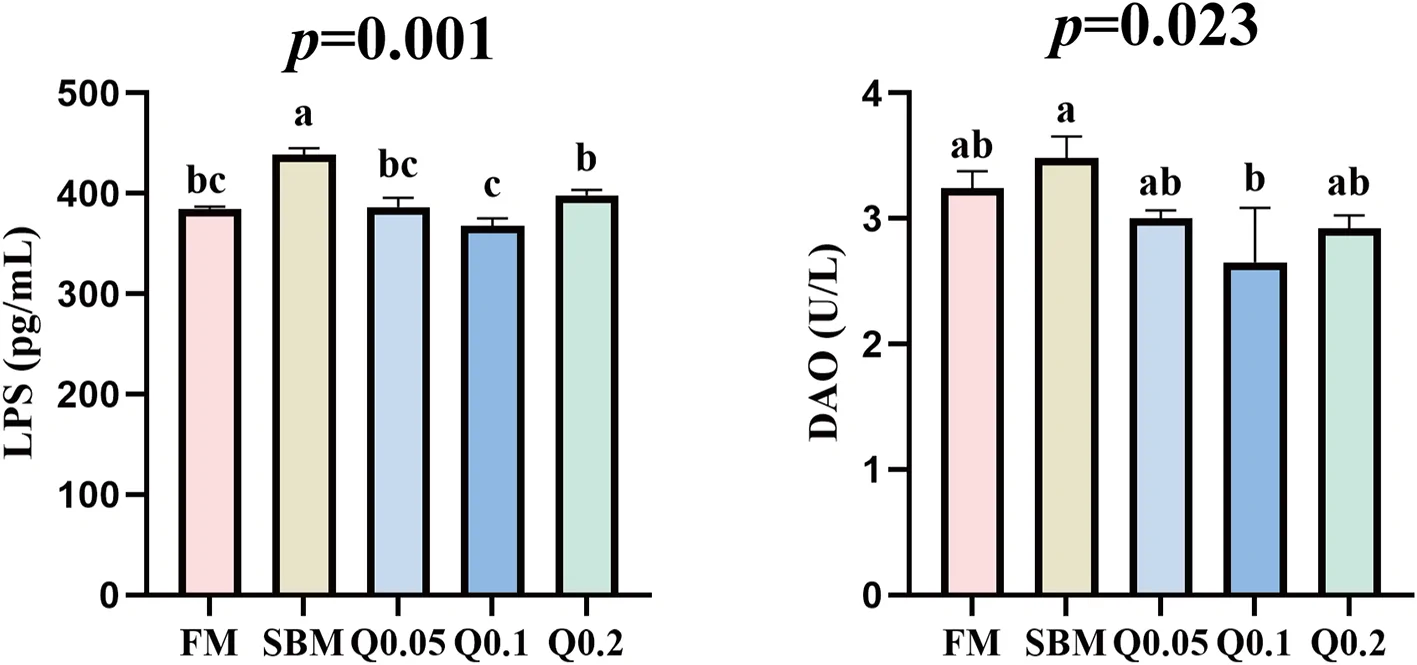
Effects of quercetin supplementation in high-soybean meal diets on gut barrier integrity of juvenile turbot. Data were expressed as mean ± SEM and analyzed by One-way ANOVA, followed by Tukey’s multiple range test (n=3). Means in the same row sharing the same superscript letter are not significantly different determined by Tukey's test (*P >*0.05). LPS, Lipopolysaccharide; DAO, Diamine oxidase.

### 3.8 Serum physio-biochemical indices

There was no significant difference in serum ALT activity among the FM and the quercetin-supplementation groups (*P* > 0.05), and ALT activity in the SBM group were significantly higher than those in the FM and Q0.2 groups (*P* < 0.05). Comparisons of SBM, Q0.1 and Q0.2 groups revealed no obvious statistical differences in serum AST activity (*P* > 0.05). However, AST activity in the SBM group were significantly higher than those in the FM and Q0.05 groups (*P* < 0.05). There were no significant differences in serum γ-GT activity among the FM, SBM, and Q0.1 groups (*P* > 0.05), and γ-GT activity in the SBM group were significantly higher than those in the Q0.05 and Q0.2 groups (*P* < 0.05). There were no significant differences in serum TG content among the FM, SBM, and Q0.2 groups (*P* > 0.05). TG content were statistically higher in the SBM group than those receiving Q0.05 and Q0.1 groups (*P* < 0.05). There were no significant differences in ALB activity and AKP activity among the different treatment groups (*P* > 0.05) (**Table 8**).

**Table 8:** Effects of quercetin supplementation in high-soybean meal diets on non-specific immune indices of juvenile turbot. ^1^.

Indices	FM	SBM	Q0.05	Q0.1	Q0.2	*p*-Value
ALB (g/L) ^2^	7.33 ± 0.75	6.64 ± 0.15	7.28 ± 0.73	6.64 ± 0.78	6.44 ± 0.46	0.819
ALT (U/L) ^2^	19.6 ± 0.44^b^	25.56 ± 1.73^a^	22.2 ± 2.03^ab^	21.8 ± 0.85^ab^	19.87 ± 0.40^b^	0.045
AST (U/L) ^2^	7.14 ± 0.26^b^	12.37 ± 0.56^a^	7.72 ± 0.53^b^	9.92 ± 1.51^ab^	9.27 ± 1.19^ab^	0.004
AKP (King unit/gprot) ^2^	3.42 ± 0.51	2.9 ± 0.11	2.39 ± 0.11	2.7 ± 0.28	2.48 ± 0.2	0.223
γ-GT (U/L) ^2^	17.62 ± 2.82^ab^	25.53 ± 2.88^a^	11.18 ± 0.59^b^	13.86 ± 1.05^ab^	13.15 ± 2.61^b^	0.010
TG (mmol/gprot) ^2^	5.45 ± 0.27^ab^	6.10 ± 0.34^a^	2.79 ± 0.25^b^	2.92 ± 0.37^b^	3.60 ± 0.92^ab^	0.013

^1^ Data were presented as mean ± SD (n=3). Means in the same row sharing the same superscript letter are not significantly different determined by Tukey's test (*P >*0.05).

^2^ ALB, Albumin; ALT, Alanine transaminase; AST, Aspartate aminotransferase; AKP, Alkaline phosphatase; γ-GT, γ-glutamyltransferase; TG, Triglycerides.

### 3.9 Histomorphology of liver tissue

The liver cells in the FM group were evenly distributed, with clear boundaries between the cell nuclei and cell membranes, in contrast, the liver cells in the SBM group exhibited nuclear dissolution or absence, and some showed vacuolization. After adding quercetin to the high-soybean-meal diet, the degree of hepatocyte vacuolization decreased compared to the SBM group. In the Q0.2 group, the dissolution or absence of hepatocyte nuclei improved, but vacuolization still persisted; whereas in the Q0.05 and Q0.1 groups, the hepatocyte nuclei were relatively distinct, and the vacuolization was significantly improved (**Figure 8**).

**Figure 8:**
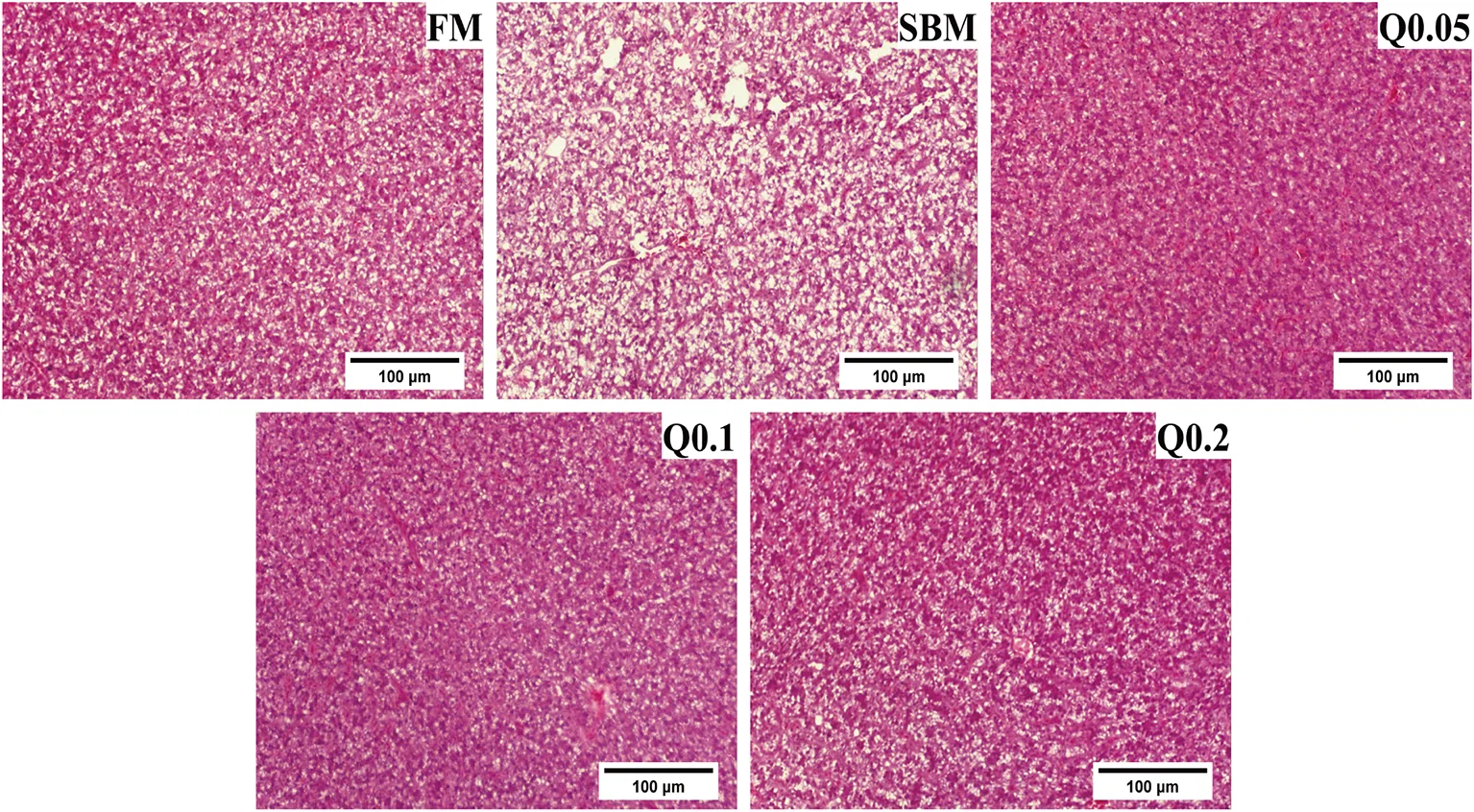
Effects of quercetin supplementation in high-soybean meal diets on hepatic histomorphology of juvenile turbot. Images of HE-stained sections (200 ×, scale bar: 100 μm) (n=3).

The FM group had a low number of lipid droplets, consisting mainly of small, scattered droplets with no large droplets, and exhibited low levels of hepatic lipid accumulation; in contrast, the SBM group had a high number of lipid droplets, consisting mainly of large, round droplets that were densely distributed and fused together, resulting in severe hepatic lipid accumulation. After the addition of quercetin, the lipid accumulation was significantly improved (**Figure 9A**). There were no significant differences among the FM, Q0.05, and Q0.2 groups (*P* > 0.05). The lipid droplet area in the Q0.1 group was significantly lower than that in the SBM, Q0.05, and Q0.2 groups (*P* < 0.05) (**Figure 9B**).

**Figure 9:**
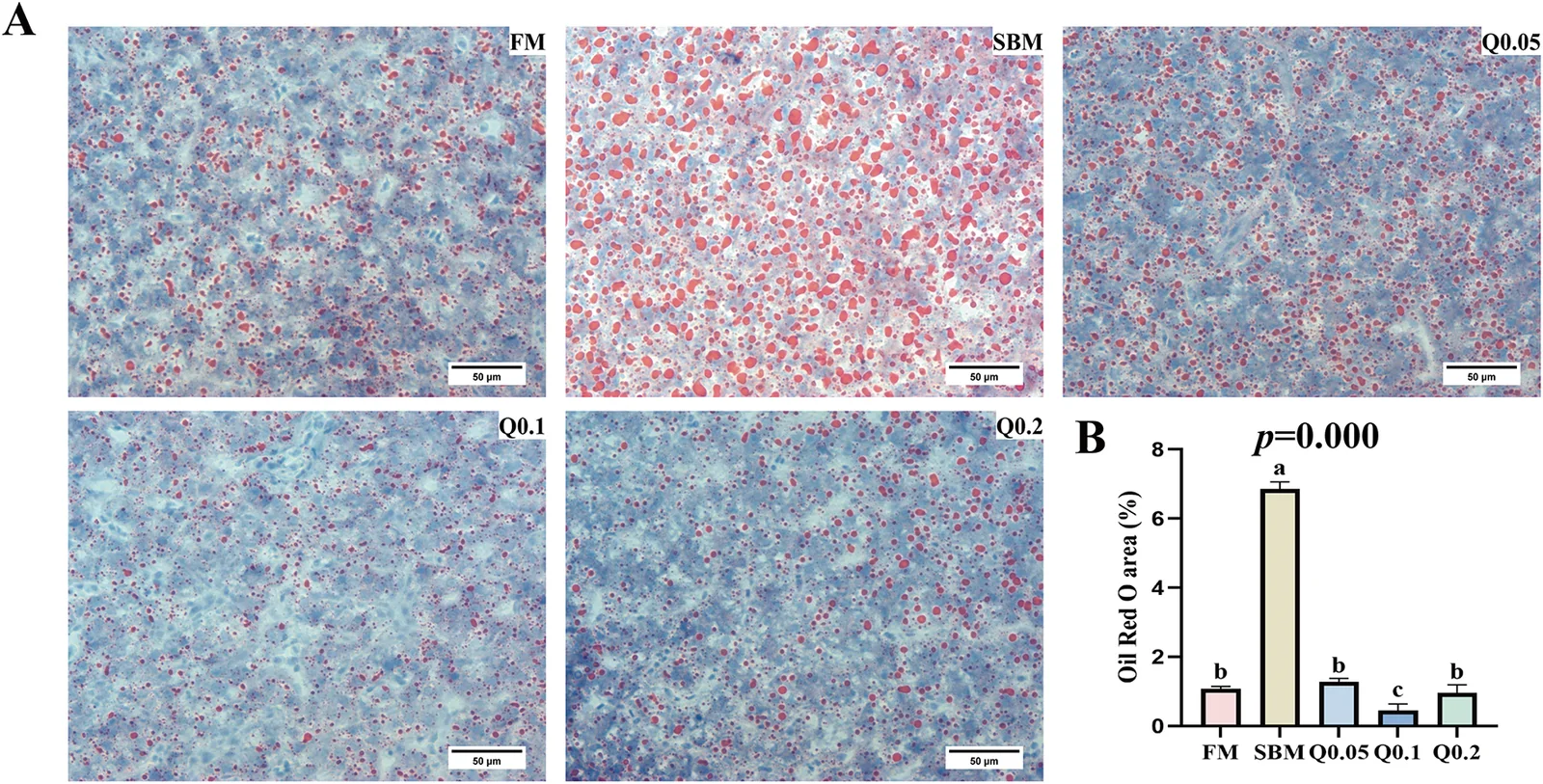
Effects of quercetin supplementation in high-soybean meal diets on hepatic lipid deposition of juvenile turbot. **(A)** Oil red o stain (400 ×, scale bar: 50 μm). **(B)** Data were presented as mean ± SD (n=3). Means in the same row sharing the same superscript letter are not significantly different determined by Tukey's test (*P >*0.05).

Transmission electron microscopy revealed that in the FM group, the endoplasmic reticulum in the liver was neatly and tightly arranged, and the mitochondrial ultrastructure remained intact, characterized by rich matrix and regularly aligned cristae. In the SBM group, the endoplasmic reticulum was swollen and fragmented, with a loose arrangement and abnormal appearance, the mitochondria were severely vacuolated and had lost their cristae. After adding quercetin to the high-soybean-meal diet, the abnormal state of the hepatic endoplasmic reticulum was significantly alleviated and largely returned to normal. The condition of the mitochondria also improved to some extent, with a greater number of autophagosomes observed compared to the FM group (**Figure 10**).

**Figure 10:**
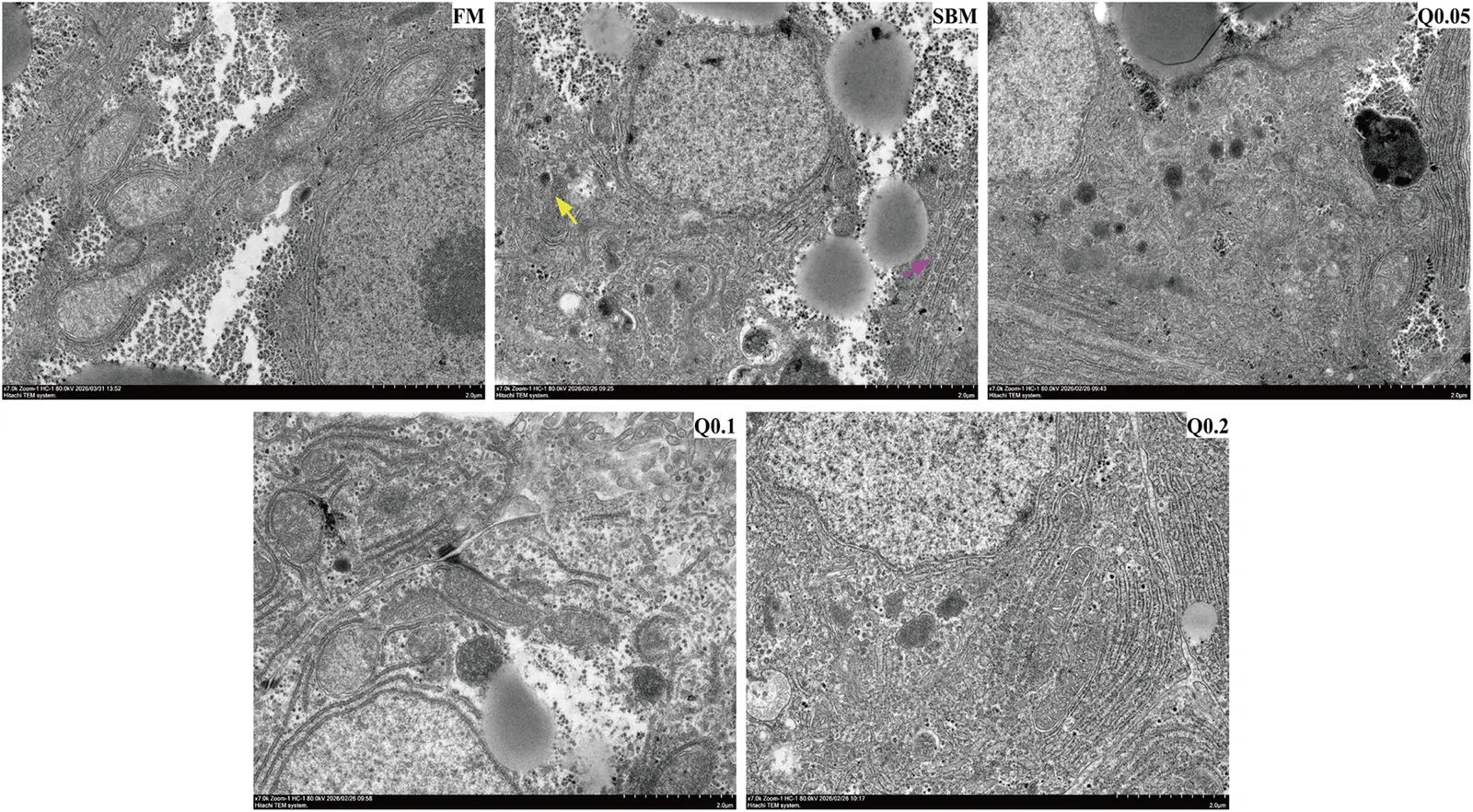
Effects of quercetin supplementation in high-soybean meal diets on hepatic ultrastructure of juvenile turbot (n=3). The yellow arrows indicated severe mitochondrial vacuolization and loss of cristae, and the magenta arrows indicated swelling and fragmentation of the endoplasmic reticulum. Scale = 2.0 μm.

ROS levels in the FM group were significantly lower than those in the SBM group, after quercetin supplementation, liver ROS levels decreased significantly compared with the SBM group (**Figure 11A**). Semi-quantitative analysis using ImageJ showed that the relative fluorescence intensity in the SBM group increased significantly. By comparison, significantly lower relative fluorescence signals were detected in fish from the Q0.05 group relative to the SBM group (*P* < 0.05) (**Figure 11B**).

**Figure 11:**
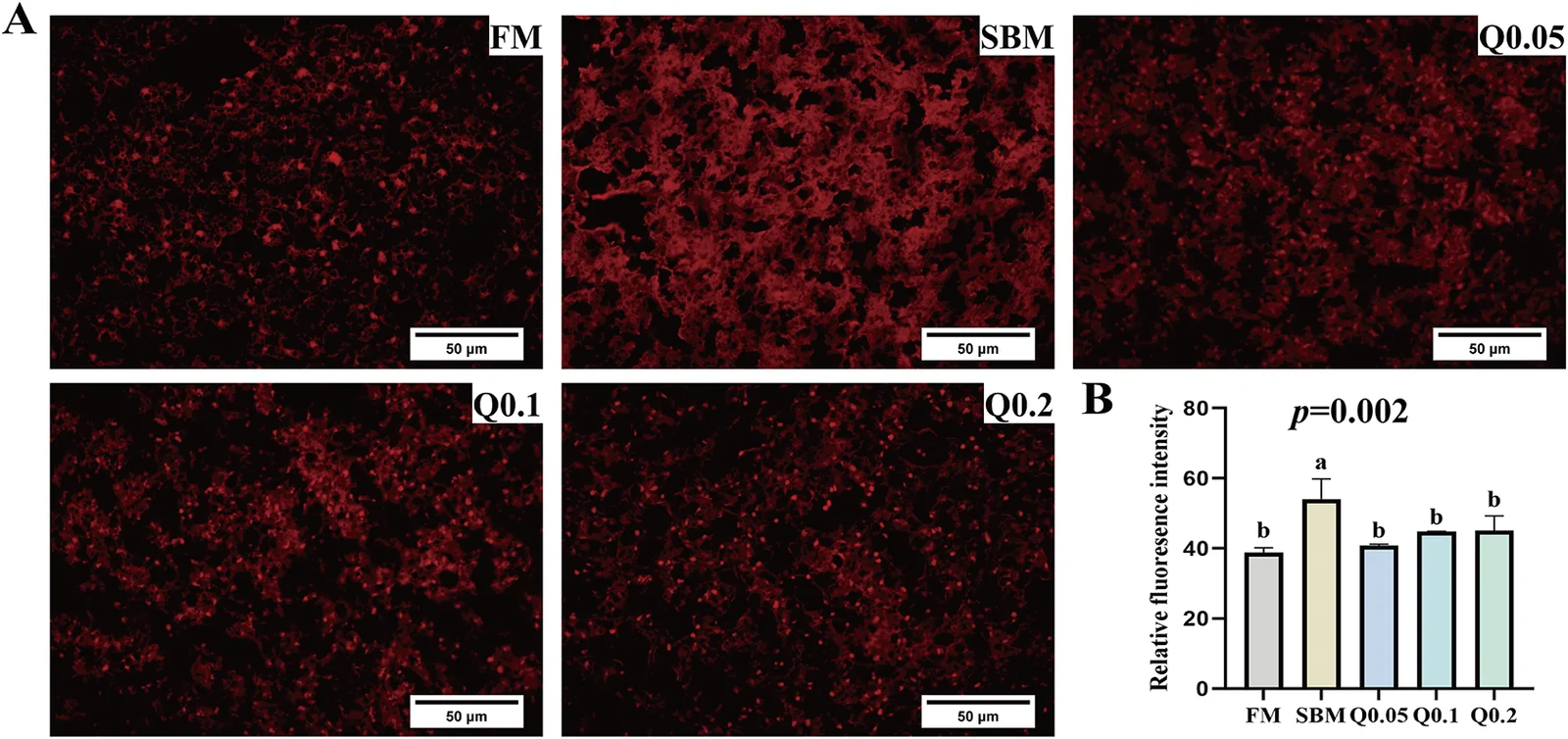
Effects of dietary quercetin supplementation on hepatic oxidative stress in juvenile scophthalmus maximus fed high soybean meal diets. **(A)** ROS accumulation in the liver was assessed via DHE staining. **(B)** Relative fluorescence intensity (n=3). Means in the same row sharing the same superscript letter are not significantly different determined by Tukey's test (*P >*0.05).

### 3.10 Gene expression in the liver

There was no significant difference in *tnf-α* mRNA expression levels among the SBM and the Q0.2 groups (*P* > 0.05). However, *tnf-α* mRNA expression levels in the SBM group were significantly higher than those in the Q0.05 and Q0.1 groups (*P* < 0.05). There were no significant differences in *tlr2* mRNA expression levels among the FM, Q0.05, and Q0.2 groups (*P* > 0.05), and *tlr2* mRNA expression levels in the SBM group were significantly higher than those in the FM and Q0.1 groups (*P* < 0.05). No statistically significant variations in *stat2* transcript abundance were found among the SBM and Q0.1 groups (*P* > 0.05), but *stat2* mRNA expression in the SBM group was significantly higher than that in the Q0.05 and Q0.2 groups (*P* < 0.05). There was no significant difference in *stat3* mRNA expression among the FM and the quercetin-supplementation groups (*P* > 0.05), but *stat3* mRNA expression in the FM and the quercetin-supplementation groups was significantly lower than that in the SBM group (*P* < 0.05). There was no significant difference in *stat5a* mRNA expression among the SBM and the quercetin-supplementation groups (*P* > 0.05), nevertheless, significantly lower *stat5a* mRNA expression were observed in fish fed the FM group compared with the SBM group (*P* < 0.05). There were no significant differences in *ifn-γ* mRNA expression levels among the different treatment groups (*P* > 0.05) (**Figure 12**).

**Figure 12:**
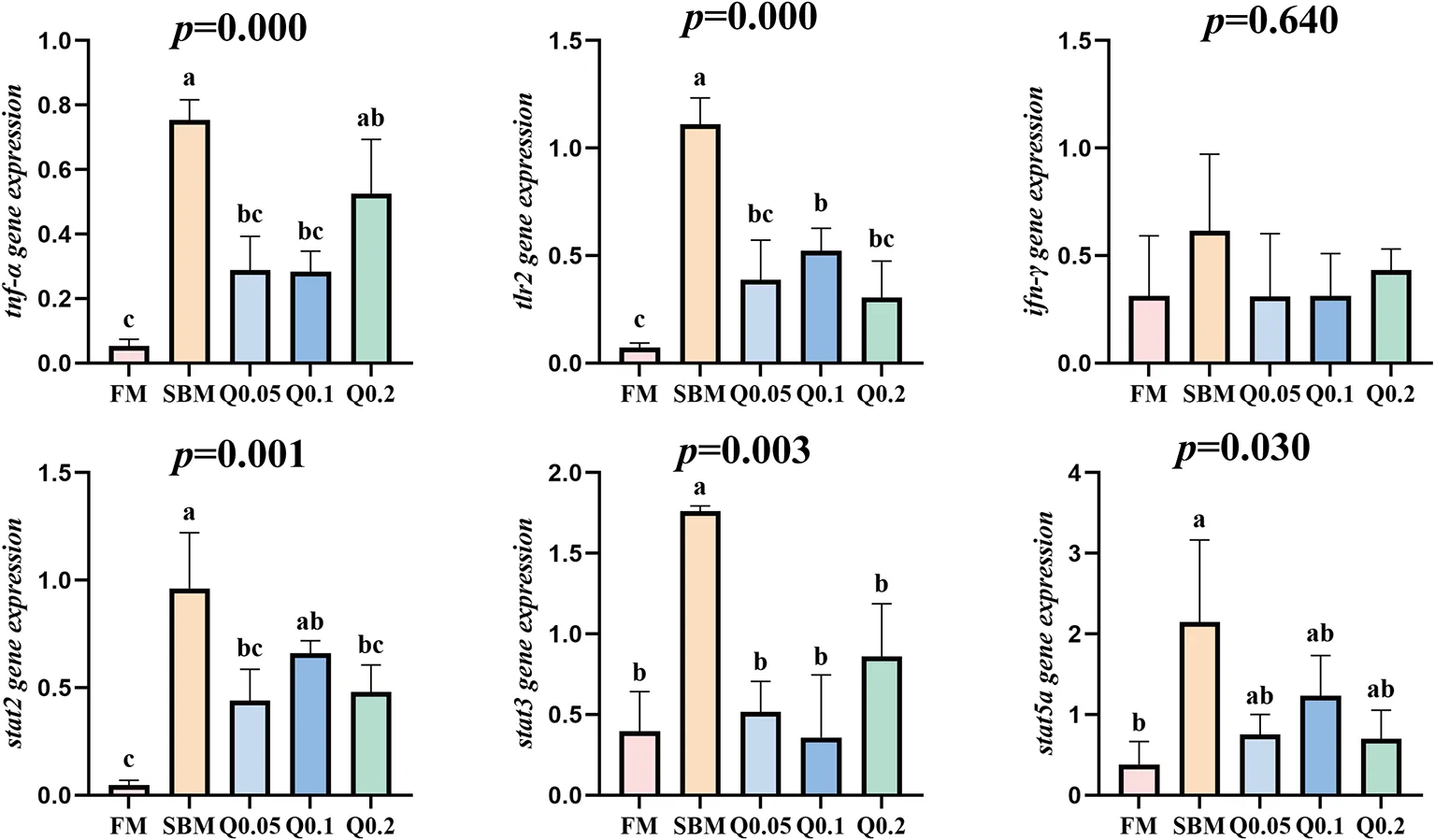
Effects of quercetin supplementation in high-soybean meal diets on hepatic inflammatory gene expression of juvenile turbot. Data were expressed as mean ± SEM and analyzed by One-way ANOVA, followed by Tukey’s multiple range test (n=3). Means in the same row sharing the same superscript letter are not significantly different determined by Tukey's test (*P >*0.05).

## 4 Discussion

The results of this study showed that, compared with the FM group, the SBM group exhibited significantly lower SGR, WGR, FE, and CF in turbot, along with a higher FCR, and HSI and VSI were significantly higher. The addition of 0.05% and 0.2% quercetin significantly increased WGR, SGR, and FE in turbot, and reducing FCR and HSI. This indicated that a high soybean meal content has an adverse effect on the growth of turbot. This may be due to soybean meal contains anti-nutritional components like trypsin inhibitors, these substances suppress the capacity of fish to take up and utilize nutrients, which consequently lowers growth indices (El-Dakar et al., 2023). Related studies have shown the growth performance of spotted bass (Zhang et al., 2025), largemouth bass (Yang et al., 2022) and red-lipped mullet (Liu et al., 2021) raised on diets high in soybean meal. According to reports, quercetin is a naturally occurring flavonoid antioxidant that primarily exhibits antioxidant, anti-inflammatory, immunomodulatory, gastroprotective, hepatoprotective, and growth-promoting effects. Shin et al. (2010) found that the weight gain rate of flounder significantly increased when fed diets supplemented with 0.25% or 0.50% quercetin. Zhai and Liu (2013) found that feeding tilapia diets containing different concentrations of quercetin (200, 400, 800, and 1600 mg/kg) resulted in improved specific growth rates, with the high-dose group showing the greatest improvement. Jia et al. (2019) reported that adding 0.8% quercetin to a high-lipid diet can improve the WGR of roundhead bream. Zhang et al. (2021) also found that dietary quercetin addition relieved triphenyltin (TPT)-induced impairment in the growth performance of zebrafish, with significant improvements in WG and SGR and a significant reduction in FCR. These previous findings align well with the present experimental outcomes. This study found that adding 0.05% and 0.2% quercetin to soybean meal-based feed significantly increased WGR, SGR and FE of turbot, and reducing the FCR and HSI, thereby promoting the growth of turbot. This study found that in turbot fed a soybean meal-based diet, SOD and GSH content decreased, and MDA content increased. In this study, adding 0.05% quercetin to soybean meal-based feed increased SOD and CAT content, and adding 0.1% quercetin significantly reduced MDA content and increased GSH and T-AOC content. Excessive lipid accumulation in fish can lead to the production of reactive oxygen species that are not cleared in a timely manner, which can cause oxidative damage to the body (Zhang et al., 2018). During the course of evolution, fish have developed an antioxidant enzyme defense system to protect their bodies from oxidative stress damage (Matés et al., 1999). MDA can serve as a marker for measuring the level of oxidative stress (Xie et al., 2020). The results of this study were consistented with those reported for largemouth bass (Yang et al., 2022). This indicated that feed with a high soybean meal content can reduce the fish’s antioxidant capacity, leading to oxidative stress damage. Quercetin functions as a powerful antioxidant, with two antioxidative moieties existing within its molecular framework: the catechol group in the B ring and the hydroxyl (-OH) group at the 3-position of the AC ring (Boots et al., 2008). It exhibited strong antioxidant activity and can effectively scavenge free radicals in the body, thereby reducing oxidative stress-induced damage (Xu et al., 2019). Existing research has verified that dietary quercetin improves the antioxidant status of black bass (Kong et al., 2022), rosy barb (Han et al., 2025) and tilapia (Khan et al., 2023), and boost the body’s antioxidant capacity. The above results are similar to those of this study. In this study, compared with the FM group, the SBM group showed significantly lower content of TP, TRY, AMS, and lipase in turbot. Following the addition of quercetin, the content of TP, AMS, lipase, and TRY activity in the turbot gut increased significantly, with the highest levels observed when 0.1% and 0.2% quercetin were added. Gut digestive enzyme activity reflected gut digestive capacity and health (Lin and Xiao, 2006). Studies by Sveier et al. (2008) on Atlantic salmon founded that trypsin inhibitors presented in soybeans bind directly to proteases in the gut, thereby reducing protease activity in the gut. Studies have shown that adding 5 mg/L of quercetin to the diet significantly increases the activity of AMS and lipase in the guts of rosy barb (Zhu et al., 2024). A study by Ghafarifarsani et al. (2022) also founded that adding 200 mg/kg of quercetin increased gut TP activity in carp. In addition, 2.5 g/kg of quercetin significantly increased the activity of AMS, lipase, and TRY in the guts of tilapia (Abdo et al., 2024). The conclusions documented in former studies match those acquired in this trial. This indicated that quercetin can enhance the digestive capacity of the fish’s guts, thereby boosting their immunity and promoting growth.

In this study, compared with the FM group, the SBM group exhibited significantly lower gut villus height, microvillus height, muscular layer thickness, and number of goblet cells in juvenile turbot. However, the addition of a suitable dosage of quercetin to the soybean meal-based diet increased gut villus height, microvillus height, muscular layer thickness, and the number of goblet cells in juvenile turbot. The height of gut villi and the thickness of the muscular layer played important roles in nutrient absorption (Abdel-Latif et al., 2020) and in enhancing growth performance (Wang et al., 2023b). Reports indicated that adding plant-based protein sources to feed can induce significant structural changes in the intestinal tissues of white bass (Pérez-Velasco et al., 2026) and flounder (Chen et al., 2011), including changes in intestinal villus height, muscle layer thickness, and the number of goblet cells. In addition, adding 0.25 g/kg of quercetin to high-soybean-meal feed significantly increased the villus height and muscle layer thickness in spotted bass (Liu et al., 2024). Feng et al. (2024) found in their study of carp that the addition of 400 mg/kg of quercetin significantly improved the morphological structure of the carp’s guts. The findings reported in previous literature are consistent with the present experimental data. This indicated that quercetin can improve the gut histological traits of juvenile turbot fed diets with soybean meal as the primary protein source, thereby promoting nutrient absorption. The results of this study showed that the expression of the gut tight junction proteins *claudin-7*, *occludin*, and *tricellulin* in juvenile turbot fed a soybean meal-based diet was significantly lower than that in the FM group. However, the addition of quercetin significantly upregulated the expression of these tight junction proteins. Studies on largemouth bass have shown that antinutritional components in soybean meal can compromise the integrity of the gut mucosa and induce gut inflammation (Rossi et al., 2021). At the same time, studies have shown that quercetin not only increases the expression of tight junction protein genes and promotes the proliferation of gut cells, but also reduces gut mucosal permeability, thereby limiting the entry of lipopolysaccharides from the gut into the bloodstream and improving the morphology of gut tissue (Suzuki and Hara, 2011). Our experimental data exhibit the same trends as those reported in the above references. This indicated that quercetin can alleviate the loosening of tight junctions in the guts and increased barrier permeability in juvenile turbot caused by high soybean meal stress, as well as the hepatic and gut inflammatory damage induced by endotoxin and antigen invasion. In this study, compared with the FM group, the SBM group showed significant downregulation of *mtor*, *bcl-2*, and *nrf-2*, and significant upregulation of *p38* and *jnk*. Following the addition of quercetin, *mtor*, *bcl-2*, and *nrf-2* were significantly upregulated, and *p38* and *jnk* were significantly downregulated. Studies have shown that soybean meal-based diets increase the gene expression of pro-inflammatory factors in the gut of turbot and decreasing the gene expression of anti-inflammatory factors (Chen et al., 2018). *mtor* is involved in the development of inflammatory bowel disease (Fallahi et al., 2021), and *jnk* can activate the expression of various genes involved in inflammation (Xu et al., 2024). *bcl-2* was a key target molecule in the apoptotic pathway, and *nrf-2* played a pivotal role in maintaining oxidative homeostasis in the body (Zhou et al., 2023). Previous published outcomes align with the present results. This indicated that soybean meal-based feed induces gut inflammation in fish, leading to a weakened ability to combat inflammation and severe gut damage. Previous studies have shown that quercetin effectively reduces cellular inflammatory factors, the addition of quercetin enhanced the anti-inflammatory capacity of the carp gut and attenuated the expression of inflammation-related genes (Zhu et al., 2024). This indicated that quercetin can enhance the anti-inflammatory capacity of the guts in juvenile turbot, thereby facilitating the repair of gut tissue. The results of this study showed that the Chao-1 index, Simpson’s index, and Shannon index were significantly lower in the SBM group compared to the FM group. Adding 0.1% quercetin to a high-soybean-meal diet increased the Chao-1 index, Simpson’s index, and Shannon index of the gut microbiota in juvenile turbot. The gut microbiota of fish played a crucial role in maintaining gut health, promoting gut development, and combating microbial invasion (Bondia-Pons et al., 2015). A study by Chen et al. (2024) showed that when largemouth bass were fed a high-soybean-meal diet, the Chao-1 index, Simpson’s index, and Shannon index all decreased significantly. A study by Liu et al. (2024) showed that adding 0.25 g/kg of quercetin significantly increased the Chao-1 index, Shannon index, and observed species richness in spotted bass. This suggested that adding an appropriate amount of quercetin to high-soybean-meal diets helped improve the alpha diversity of the gut microbiota in juvenile turbot. At the same time, the Venn diagram showed that the Q0.1 group had the highest number of unique OTUs, indicating the emergence of a large number of new species in the gut microbiota, which may be a quercetin-induced unique microbial community. Additionally, the species abundance bar chart and LEfSe analysis reveal that, at the phylum level, the gut microbiota of juvenile turbot was primarily composed of the Pseudomonadota, Bacillota, Bacteroidota and Halobacteriota. The addition of quercetin increased the relative abundance of Pseudomonadota in the gut of juvenile turbot and decreasing the relative abundances of Bacillota, Bacteroidota and Halobacteriota. This was similar to the gut microbiota composition of the yellow catfish (Wang et al., 2021). Studies have shown that the Pseudomonadota is a characteristic microbial group associated with gut inflammation (Shin et al., 2015), the Bacteroidota can serve as an indicator of gut health in animals (Turnbaugh et al., 2009), and the *Firmicutes* and *Actinobacteria* played important roles in promoting nutrient absorption and metabolism in fish (Mountfort et al., 2002; Bradlow, 2014). Prior relevant findings are in line with the results of this investigation. The results indicated that a high-soybean-meal diet induces an imbalance in the gut microbiota of juvenile turbot and increases the risk of inflammation. However, the addition of quercetin increased the abundance of dominant bacterial phyla, thereby improving gut digestive function and gut health.

LPS and DAO are indicators of gut damage. In this study, compared with the FM group, serum LPS and DAO levels in SBM-fed halibut were significantly elevated. The addition of quercetin to the soybean meal-based diet significantly reduced serum LPS and DAO levels, and the lowest levels of serum LPS and DAO were observed in halibut fed a diet supplemented with 0.1% quercetin. A wide array of anti-nutrient compounds can be detected in soybean meal according to previous literature, including soybean antigens, lectins, and phytic acid (Francis et al., 2001). These antinutritional factors can disrupt the integrity of the intestinal mucosa and trigger intestinal inflammation (Arriaga-Hernandez et al., 2021). LPS is one of the key mechanisms underlying Gram-negative bacterial infections, leading to inflammatory damage in the gut (Ling et al., 2026). Villus epithelial cells distributed on the surface of intestinal mucosa secrete a highly catalytic intracellular enzyme known as DAO. Under normal conditions, it is rarely detected in serum. However, when the gut mucosal barrier is compromised, a rapid elevation can be observed in its serum activity (Liu et al., 2024). This indicated that a high-soybean-meal diet leads to impaired gut structural integrity and increased gut mucosal permeability in turbot, and also inducing an inflammatory response in the gut. At this point, LPS and DAO enter the liver via the bloodstream, triggering an inflammatory response in the liver. *In vitro* studies have shown that quercetin can inhibit LPS-induced production of cyclooxygenase (COX) and lipoxygenase (LOX) (Chen et al., 1986). In addition, a study by Liu et al. (2024) showed that quercetin can alleviate soybean meal-triggered detrimental impacts on the intestines of spotted bass by enhancing the defensive function of gut mucosal barrier. The above results are similar to those of this study. This suggested that quercetin improved the morphology of gut tissue, reduced gut mucosal permeability, and played a role in repairing the gut mucosal barrier. Enhanced intestinal digestive enzyme activities mediated by quercetin relieved hepatic impairment induced by high soybean meal inclusion, maintaining the structural integrity of gut tissues, upregulating the expression of tight junction proteins, suppressing excessive inflammatory responses, and restoring gut microbiota diversity. It reduced the release of LPS and DAO, thereby interrupting the cascade of events at multiple critical points along the gut-liver axis.

In this study, compared with the FM group, the serum activity of AST, ALT, γ-GT and content of TG were significantly higher in the SBM group of halibut. The results indicated that adding 0.05% and 0.2% quercetin to soybean meal-based diets significantly reduced the activity of AST, ALT, γ-GT and content of TG in halibut, and simultaneously increasing ALB activity. Studies have shown that when liver cells are damaged, serum activity of AST and ALT rise significantly (Richard et al., 1977). Elevated serum TG content are considered a risk indicator for lipid accumulation and lipid metabolism disorders (Ming et al., 2023). Studies have shown that feeding a high-soybean diet induces elevated activity of AST, ALT, and content of TG in pufferfish (Ye et al., 2019). This indicated that soybean meal-based feed induces liver cell damage in fish, leading to liver inflammation, and simultaneously disrupting lipid anabolism in the liver, causing lipids to accumulate in liver parenchymal cells. Studies by Ghafarifarsani et al. (2022) and Xin et al. (2024) have shown that adding 800 mg/kg and 400 mg/kg of quercetin to feed can reduce AST and ALT activity in carp. Shin et al. (2010) founded that quercetin reduces TG content in the serum of flounder, with the effect being positively correlated with the dose and duration of quercetin administration. In addition, Zhao et al. (2021) also founded that the addition of quercetin reduced activity of AST, ALT, γ-GT and content of TG in zebrafish, thereby protecting liver function and reducing alcohol-induced steatosis in zebrafish hepatocytes. Previous research yields findings consistent with this trial. The results indicated that quercetin alleviates high-soybean-meal-induced liver inflammation and lipid accumulation in turbot via the enterohepatic axis, improves the health of the liver in juvenile turbot, and promotes the breakdown and utilization of lipid by the liver. This study demonstrated through liver tissue sections that soybean meal-based feed induced liver cell damage in juvenile turbot, such as vacuolization of hepatocytes and nuclear lysis or disappearance. Furthermore, the soybean meal-based feed caused severe lipid deposition in the livers of juvenile turbot, resulting in obvious steatosis. Concurrently, the endoplasmic reticulum in the liver was swollen and ruptured, with loose and abnormal morphology, and the mitochondria exhibited severe vacuolization. However, the addition of 0.1% quercetin to the soybean meal-based diet for turbot alleviated the aforementioned symptoms. Studies have shown that as the amount of soybean meal added to feed increases, liver function in fish decreases significantly (Ostaszewska et al., 2005). Studies on stonefish (Fuentes-Quesada et al., 2018) and flounder (Chen et al., 2011) have both demonstrated that as the level of soy protein replacing fish meal in the diet increases, significant lesions appear in the liver tissue. This may be due to damage to the liver caused by soluble antinutritional factors (saponins and non-starch polysaccharides) in soy protein sources (Refstie et al., 2005). The results of a study by Ashour et al. (2023) in mice showed that quercetin significantly improved lipid deposition and fibrotic lesions in the livers of rats with nonalcoholic steatohepatitis and reduced inflammatory cell infiltration. In addition, this study demonstrated through liver ROS sections that soybean meal-based feed induced oxidative stress in the livers of juvenile turbot, accompanied by a significant increase in fluorescence intensity. After adding quercetin, the oxidative stress was markedly alleviated, with the most significant improvement observed at a quercetin concentration of 0.05%. Previous studies have shown that adding 400 mg/kg of quercetin to black bass feed can significantly reduce liver oxidative levels and fluorescence intensity (Wei et al., 2025). In this study, compared with the FM group, the JAK-STAT signaling pathway was activated in the livers of juvenile turbot in the SBM group. *stat2*, *stat3*, and *stat5a* were significantly upregulated in the soybean meal group, and the expression levels of *tnf-α*, *tlr2*, and *ifn-γ* also increased significantly. Following the addition of quercetin, the expression of *tnf-α*, *tlr2*, *ifn-γ*, *stat2*, *stat3*, and *stat5a* were significantly inhibited and downregulated. *tnf-α* and *ifn-γ* are key immune-related pro-inflammatory cytokines in fish (Ibrahim et al., 2021; Rozario et al., 2025). Inflammatory cues consistently act on the JAK-STAT pathway, a ubiquitous intracellular signal transduction system (Li et al., 2026). In studies on grass carp, water extracts of soybean meal have been shown to induce oxidative stress in primary liver cells of grass carp and exacerbate liver cell damage and necrosis (Bai et al., 2010). Similar experimental results have been reported in black fish (Kong et al., 2022) and tilapia (Ibrahim et al., 2021), indicating that the addition of quercetin can enhance liver immunity and disease resistance. Our study display identical variation rules to those mentioned above. This suggested that gut damage caused by high-soybean-meal diets triggers a cascade of immune and inflammatory responses via the gut-liver axis, disrupting the homeostasis of the gut-liver circulation and ultimately leading to liver damage (Duan et al., 2025).

## 5 Conclusion

Dietary quercetin supplementation in diets with 40% fish meal replacement by soybean meal significantly enhanced antioxidant capacity and protected gut and liver health in turbot, with concomitant improvements in growth performance. The gut-liver axis was possibly involved in this regulatory process. The optimal dietary quercetin supplementation level was 0.2% in the present study. Limitations of the present study should be acknowledged. Although our results indicated that dietary quercetin improved the antioxidant capacity and gut and hepatic health of turbot, this study could not establish a causal role of the gut-liver axis in alleviating soybean meal-induced gut and hepatic impairment. Mechanistic verification via gut-liver axis-related intervention or rescue experiments was not performed in the current trial. Future research will carry out targeted intervention studies to further dissect the causal mechanism underlying the gut-liver axis-mediated protective effects of quercetin.

## Data Availability

The data analyzed in this study is subject to the following licenses/restrictions: Not Applicable. Requests to access these datasets should be directed to Zhijie Dan, zjdan@jou.edu.cn.

## References

[B1] Abd El-Aziz A. Ghanima M. A. Kamal M. Aldhalmi A. K. Khan M. M.H. El-Hack M. E. A. . (2025). Quercetin as a bioactive feed additive: nutritional, antioxidant, and gut health implications in poultry and rabbits. Vet. Anim. Sci. 16, 100487. doi: 10.1016/j.vas.2025.100487 40740308 PMC12308014

[B2] Abdel-Latif H. M. Abdel-Tawwab M. Khafaga A. F. Dawood M. A. O. (2020). Dietary oregano essential oil improved the growth performance via enhancing the gut morphometry and hepato-renal functions of common carp (*Cyprinus carpio* L.) fingerlings. Aquaculture 526, 735432. doi: 10.1016/j.aquaculture.2020.73543242574925

[B3] Abdo H. S. Mohammady E. Y. Tonsy H. D. Ibrahim A. Hassaan M. S. (2024). The potential synergistic action of quercetin and/or Pediococcus acidilactici on Nile tilapia, *Oreochromis niloticus* performance. Aquaculture 581, 11. doi: 10.1016/j.aquaculture.2023.74035342574925

[B4] Anand A. Arulmoli R. Parasuraman S. (2016). Overviews of biological importance of quercetin: a bioactive flavonoid. Pharmacogn. Rev. 10, 84–89. doi: 10.4103/0973-7847.194044 28082789 PMC5214562

[B5] Arriaga-Hernandez D. Hernandez C. Martinez-Montano E. Ibarra-Castro L. Lizárraga-Velázquez E. Leyva-López N. . (2021). Fish meal replacement by soybean products in aquaculture feeds for white snook, *Centropomus Viridis*: effect on growth, diet digestibility, and digestive capacity. Aquaculture 530, 735823. doi: 10.1016/j.aquaculture.2020.73582342574925

[B6] Ashour H. Rashed L. A. Hassanein R. Aboulhoda B. E. Ebrahim H. A. Elsayed M. H. . (2023). Thymoquinone and quercetin protect against hepatic steatosis in association with SIRT1/AMPK stimulation and regulation of autophagy, perilipin-2, and cytosolic lipases. Arch. Physiol. Biochem. 129, 268–281. doi: 10.1080/13813455.2022.2134423 36264662

[B7] Bai H. Hai C. Xi M. Liang H. Liu R. (2010). Protective effect of maize silks (*Maydis stigma*) ethanol extract on radiation-induced oxidative stress in mice. Plant Foods Hum. Nutr. 65, 271–276. doi: 10.1007/s11130-010-0172-6 20526679

[B8] Bondia-Pons I. Hyötyläinen T. Orešič M. (2015). Role of Microbiota in Regulating Host Lipid Metabolism and Disease Risk. In: Kochhar S. Martin F. P. (eds) Metabonomics and Gut Microbiota in Nutrition and Disease. Molecular and Integrative Toxicology. London: Springer, 235–260. doi: 10.1007/978-1-4471-6539-2_12

[B9] Boots A. W. Haenen G. R. Bast A. (2008). Health effects of quercetin: from antioxidant to nutraceutical. Eur. J. Pharmacol. 585, 325–337. doi: 10.1016/j.ejphar.2008.03.008 18417116

[B10] Bradlow H. L. (2014). Obesity and the gut microbiome: pathophysiological aspects. Hormone Mol. Biol. Clin. Invest. 17, 53–61. doi: 10.1515/hmbci-2013-0063 25372730

[B11] Chen C. J. Chuang Y. C. You S. L. Xu W. Liu F. Z.G. Zhang W. B. . (1986). A retrospective study on malignant neoplasms of bladder, lung and liver in blackfoot disease endemic area in Taiwan. Br. J. Cancer 53, 399–405. doi: 10.1038/bjc.1986.65 3964542 PMC2001352

[B12] Chen W. Ai Q. Mai K. Lin T. M. Wu H. Y. (2011). Effects of dietary soybean saponins on feed intake, growth performance, digestibility and gut structure in juvenile Japanese flounder (*Paralichthys olivaceus*). Aquaculture 318, 95–100. doi: 10.1016/j.aquaculture.2011.04.05042574925

[B13] Chen W. Gao S. Chang K. Zhang Z. Y. Zhao N. Huang Y. C. . (2024). Partial fishmeal replacement by soybean meal induces fish growth retardation and gut inflammation via gut mucosal barrier dysfunction and dysbiosis in largemouth bass. Anim. Feed Sci. Technol. 316, 116067. doi: 10.1016/j.anifeedsci.2024.11606742574925

[B14] Chen Z. C. Liu Y. Li Y. X. Yang P. Hu H. B. Yu G. J. . (2018). Dietary arginine supplementation mitigates the soybean meal induced enteropathy in juvenile turbot, *Scophthalmus maximus* L. Aquacult. Res. 49, 1535–1545. doi: 10.1111/are.13608

[B15] Dan Z. J. Zhang W. C. Zheng J. C. Gong Y. Cui K. Mai K. S. . (2022). Effects of fishmeal substitution by four fermented soybean meals on growth, antioxidant capacity and inflammation responses of turbot juveniles (*Scophthalmus maximus* L.). Aquaculture 560, 738414. doi: 10.1016/j.aquaculture.2022.73892742574925

[B16] Doñate C. Balasch J. C. Callol A. Bobe J. Tort L. Mac K. S. (2010). The effects of immunostimulation through dietary manipulation in the rainbow trout; evaluation of mucosal immunity. Mar. Biotechnol. 12, 88–99. doi: 10.1007/s10126-009-9203-4 19609615

[B17] Duan M. Liu J. Xu H. Xie Y. Guo W. Hui Y. . (2025). Cyhalofop-butyl induced hepatotoxicity of zebrafish through disturbing the gut-liver axis. Microchem. J. 210, 112912. doi: 10.1016/j.microc.2025.11291242574925

[B18] El-Dakar A. Y. Elgamal A. A. Amer M. A. B. Mohammed A. S. Abdel-Aziz M. F. (2023). Evaluation of fermented soybean meal by Bacillus subtilis as an alternative to fishmeal on the growth, and physiological status of Nile tilapia *Oreochromis niloticus* fingerlings. Heliyon 9, 9. doi: 10.1016/j.heliyon.2023.e19602 37809758 PMC10558826

[B19] Fallahi F. Borran S. Ashrafizadeh M. Zarrabi A. Pourhanifeh M. H. Khaksary M. M. . (2021). Curcumin and inflammatory bowel diseases: From *in vitro* studies to clinical trials. Mol. Immunol. 130, 20–30. doi: 10.1016/j.molimm.2020.11.016 33348246

[B20] Feng H. Ping K. Yang Y. Liu Z. J. Song Q. M. Chen S. . (2024). Quercetin alleviates difenoconazole-induced growth inhibition in carp through gut-brain axis. Pestic. Biochem. Physiol. 204, 106066. doi: 10.1016/j.pestbp.2024.106066 39277382

[B21] Francis G. Makkar H. Becker K. (2001). Antinutritional factors present in plant-derived alternate fish feed ingredients and their effects in fish. Aquaculture 199, 197–227. doi: 10.1016/S0044-8486(01)00526-9

[B22] Fuentes-Quesada J. P. Viana M. T. Rombenso A. N. Guerrero-Renteríaa Y. Nomura-Solísc M. Gomez-Calle V. . (2018). Enteritis induction by soybean meal in *Totoaba macdonaldi* diets: effects on growth performance, digestive capacity, immune response and distal intestine integrity. Aquaculture 495, 78–89. doi: 10.1016/j.aquaculture.2018.05.02542574925

[B23] Gao Y. Wang S. Feng G. Tang H. Ren Y. W. Yang Y. . (2026). Chitosan oligosaccharide enhances growth and immune response in *Siniperca chuatsi* cultured in recirculating aquaculture systems (RAS) by activating MAPK/NF-κB signaling in the gut-liver axis. Aquacult. Rep. 48, 103558. doi: 10.1016/j.aqrep.2026.10355842574925

[B24] Ghafarifarsani H. Hoseinifar S. H. Javahery S. Doan H. V. (2022). Effects of dietary vitamin C, thyme essential oil, and quercetin on the immunological and antioxidant status of common carp (*Cyprinus carpio*). Aquaculture 553, 738053. doi: 10.1016/j.aquaculture.2022.73805342574925

[B25] Ghosh S. S. Jing W. Yannie P. J. Ghosh S. (2020). Intestinal barrier dysfunction, LPS translocation, and disease development. J. Endocr. Soc 2, 2. doi: 10.1210/jendso/bvz039 32099951 PMC7033038

[B26] Han M. M. Zhu X. H. Li D. M. Si Q. Zhu T. Zhou Z. H. . (2025). Quercetin and taxifolin enhance immunity in Chinese sucker (*Myxocyprinus asiaticus*) and increase its resistance to Aeromonas hydrophila. Comp. Biochem. Physiol. Part. D. Genomics Proteomics 54, 101369. doi: 10.1016/j.cbd.2024.101369 39644864

[B27] Hayes M. (2023). Maximizing use of pelagic capture fisheries for global protein supply: potential and caveats associated with fish and co-product conversion into value-add ingredients. Global Challenges 7, 2200098. doi: 10.1002/gch2.202200098 37205930 PMC10190613

[B28] Hoseini S. M. Adineh H. Kulikov E. V. Vatnikov Y. A. Telezhenkova A. I. Yousef M. . (2025). Effects of dietary autolyzed yeast and quercetin on growth performance, antioxidant and immunological parameters, and resistance to heat stress in rainbow trout. Aquaculture 600, 742257. doi: 10.1016/j.aquaculture.2025.74225742574925

[B29] Ibrahim D. Kishawy A. Khater S. Khalifa E. Ismail T. A. Mohammed H. A. . (2021). Interactive effects of dietary quercetin nanoparticles on growth, flesh antioxidant capacity and transcription of cytokines and Aeromonas hydrophila quorum sensing orchestrating genes in Nile tilapia (*Oreochromis niloticus*). Fish. Shellfish Immunol. 119, 478–489. doi: 10.1016/j.fsi.2021.10.034 34699975

[B30] Jia E. T. Yan Y. N. Zhou M. Li X. F. Jiang G. Z. Liu W. B. . (2019). Combined effects of dietary quercetin and resveratrol on growth performance, antioxidant capability and innate immunity of blunt snout bream (*Megalobrama amblycephala*). Anim. Feed Sci. Technol. 256, 114268. doi: 10.1016/j.anifeedsci.2019.11426842574925

[B31] Judith A. W. Warmbrunn M. V. Max N. Max N. Karine C. (2020). Nonalcoholic lipidty liver disease: modulating gut microbiota to improve severity? Gastroenterology 158, 1881–1898. doi: 10.1053/j.gastro.2020.01.049 32044317

[B32] Kandemir K. Tomas M. McClements D. J. Capanoglu E. (2022). Recent advances on the improvement of quercetin bioavailability. Trends Food Sci. Technol. 119, 192–Max, N., Karine, C.200. doi: 10.1016/j.tifs.2021.11.03242574925

[B33] Khan T. Lipidima M. Shah S. Z. Khan N. Maryam Ali W. . (2023). Quercetin supplementation in the diet of *Labeo rohita*: effects on growth, proximate composition, antioxidative indices and immunity. Anim. Feed Sci. Technol. 303, 115699. doi: 10.1016/j.anifeedsci.2023.11569942574925

[B34] Kobori M. Takahashi Y. Akimoto Y. Sakurai M. Matsunaga I. Nishimuro H. . (2015). Chronic high intake of quercetin reduces oxidative stress and induces expression of the antioxidant enzymes in the liver and visceral adipose tissues in mice. J. Funct. Foods 15, 551–560. doi: 10.1016/j.jff.2015.04.00642574925

[B35] Kong Y. X. Tian J. X. Niu X. T. Li M. Kong Y. D. Li R. M. . (2022). Effects of dietary quercetin on growth, antioxidant capacity, immune response and immune-related gene expression in snakehead fish, *Channa argus*. Aquacult. Rep. 26, 101314. doi: 10.1016/j.aqrep.2022.10131442574925

[B36] Li N. He Z. Wang J. Ni G. Hang Y. Li K. . (2025). Gut flora and liver metabolism molecular mechanism on growth and health in Japanese eel black fry (*Anguilla japonica*) fed with fermented soybean meal replacing soybean meal and fish meal. Aquacult. Rep. 43, 102889. doi: 10.1016/j.aqrep.2025.10288942574925

[B37] Li Z. X. Li D. Li X. Gong Y. C. Feng W. W. Chen Y. . (2026). Environmental concentrations of Acetamiprid exposure induced innate immunotoxicity in zebrafish larvae via crosstalk between the TLR4/MYD88/NF-κB and JAK-STAT signaling pathways. Chem. Biol. Interact. 429, 111969. doi: 10.1016/j.cbi.2026.111969 41679451

[B38] Li M. Y. Zhu X. M. Tian J. X. Liu M. Wang G. Q. (2019). Dietary flavonoids from Allium mongolicum Regel promotes growth, improves immune, antioxidant status, immune-related signaling molecules and disease resistance in juvenile northern snakehead fish (*Channa argus*). Aquaculture 501, 473–481. doi: 10.1016/j.aquaculture.2018.12.01142574925

[B39] Lin Y. Xiao Q. Z. (2006). Dietary glutamine supplementation improves structure and function of intestine of juvenile Jian carp (*Cyprinus carpio var*. Jian). Aquaculture 256, 389–394. doi: 10.1016/j.aquaculture.2006.02.01142574925

[B40] Ling C. Li Y. Fang J. R. Dong Z. M. Zhang C. M. Ye H. . (2026). Supplementation of pectic oligosaccharide alleviates lipopolysaccharide-induced gut inflammatory damages and impairment of growth performance in broilers. Anim. Nutr. 25, 186–195. doi: 10.1016/j.aninu.2025.08.013 41938615 PMC13049288

[B41] Liu T. Han T. Wang J. T. Liu T. Bian P. Wang Y. B. . (2021). Effects of replacing fish meal with soybean meal on growth performance, feed utilization and physiological status of juvenile redlip mullet Liza haematocheila. Aquacult. Rep. 20, 100756. doi: 10.1016/j.aqrep.2021.10075642574925

[B42] Liu X. W. Mai K. S. Ai Q. LiuFu Z. G. (2015). Dietary protein requirement of juvenile turbot (*Scophthalmus maximus* L.). J. Ocean Univ. China 14, 325–328. 42877449

[B43] Liu L. Zhao Y. Huang Z. . (2024a). Evaluation of quercetin in alleviating the negative effects of high soybean meal diet on spotted sea bass *Lateolabrax maculatus*. Fish Shellfish Immunol. 150, 17. doi: 10.1016/j.fsi.2024.109607 38719096

[B44] Liu L. Zhao Y. Huang Z. . (2024b). High soybean dietary supplementation with quercetin improves antioxidant capacity of spotted sea bass *Lateolabrax maculatus*. Aquacult. Rep. 39, 102429. doi: 10.1016/j.aqrep.2024.10242942574925

[B45] Luan N. Yang Q. Sang W. Liao Z. X. Hung T. C. Zuo J. L. . (2026). Alleviating effects of Lactobacillus rhamnosus on microcystin-LR-induced hepatotoxicity in zebrafish through regulation of gut metabolism: insights into the gut-liver axis and hypothalamic-pituitary-thyroid axis. J. Environ. Sci. 167, 201–216. doi: 10.1016/j.jes.2025.12.019 42498356

[B46] Ma A. Huang Z. Wang X. A. Xu Y. H. Guo X. L. (2021). Identification of quantitative trait loci associated with upper temperature tolerance in turbot, Scophthalmus maximus. Sci. Rep. 11, 21920. doi: 10.1038/s41598-021-01062-3 34753974 PMC8578632

[B47] Matés J. M. Pérez-Gómez C. De Castro I. N. (1999). Antioxidant enzymes and human diseases. Clin. Biochem. 32, 595–603. doi: 10.1016/S0009-9120(99)00075-2 10638941

[B48] Ming J. H. Wang T. Wang T. H. Ye J. Y. Zhang Y. X. Yang X. . (2023). Effects of dietary berberine on growth performance, lipid metabolism, antioxidant capacity and lipometbolism-related genes expression of AMPK signaling pathway in juvenile black carp (*Myiopharyngodon piceus*) fed high-lipid diets. Fish. Physioligy Biochemis 49, 769–786. doi: 10.1007/s10695-022-01143-8 36418662

[B49] Mountfort D. O. Campbell J. Clements K. D. (2002). Hindgut fermentation in three species of marine herbivorous fish. Appl. Environ. Microbiol. 68, 1374–1380. doi: 10.1128/aem.68.3.1374-1380.2002 11872490 PMC123746

[B50] Ostaszewska T. Dabrowski K. Palacios M. E. Olejniczak M. Wieczorek M. (2005). Growth and morphological changes in the digestive tract of rainbow trout (*Oncorhynchus mykiss*) and pacu (*Piaractus mesopotamicus*) due to casein replacement with soybean proteins. Aquaculture 245, 273–286. doi: 10.1016/j.aquaculture.2004.12.00542574925

[B51] Pérez-Velasco R. Gómez-Gil B. Chávez-Sánchez M. C. Mesina-Peña A. Flores-Méndez L. C. Martínez-Brown J. M. . (2026). Effect of replacing fish meal with conventional or fermented soybean meal on gut morphology, microbiota, and oxidative stress biomarkers in the white snook (*Centropomus viridis*). Aquacult. Rep. 46, 103269. doi: 10.1016/j.aqrep.2025.10326942574925

[B52] Porras D. Nistal E. Martínez-Flórez S. Pisonero-Vaquero S. Olcoz J. L. Jover R. . (2017). Protective effect of quercetin on high-lipid diet-induced non-alcoholic lipidty liver disease in mice is mediated by modulating gut microbiota imbalance and related gut-liver axis activation. Free Radical Biol. Med. 102, 188–202. doi: 10.1016/j.freeradbiomed.2016.11.037 27890642

[B53] Refstie S. Sahlström S. Bråthen E. Baeverfjord G. Krogedal P. (2005). Lactic acid fermentation eliminates indigestible carbohydrates and antinutritional factors in soybean meal for Atlantic salmon (*Salmo salar*). Aquaculture 246, 331–345. doi: 10.1016/j.aquaculture.2005.01.00142574925

[B54] Richard J. Strance C. B. Schreck B. (1977). Corticoid stress response to handling and temperature in salmonids. Trans. Am. Fish. Soc 106, 213–218. doi: 10.1577/1548-8659(1977)106<213:CSRTHA>2.0.CO;2

[B55] Rossi W. Allen K. M. Habte-Tsion H. M. Meesala K. M. (2021). Supplementation of glycine, prebiotic, and nucleotides in soybean mealbased diets for largemouth bass (*Micropterus salmoides*): effects on production performance, whole-body nutrient composition and retention, and gut histopathology. Aquaculture 532, 736031. doi: 10.1016/j.aquaculture.2020.73603142574925

[B56] Rozario P. Lim Y. S. Ding S. L. Firdaus M. J. Wearne S. Wong B. H.S. . (2025). IFNγ priming enables NLRP3 inflammasome activation in human keratinocytes *in vitro*. J. Invest. Dermatol. 146, 1851–1866. doi: 10.1016/j.jid.2025.11.019 41389956

[B57] Shin N. R. Whon T. W. Bae J. W. (2015). *Proteobacteria*: microbial signature of dysbiosis in gut microbiota. Trends Biotechnol. 33, 496–503. doi: 10.1016/j.tibtech.2015.06.011 26210164

[B58] Shin H. S. Yoo J. H. Min T. S. Lee J. Choi C. Y. (2010). Effect of quercetin on the activity and mRNA expression of antioxidant enzymes and physiological responses in olive flounder (*Paralichthys olivaceus*) exposed to cadmium. Asian-Australas. J. Anim. Sci. 23, 742–749. doi: 10.5713/ajas.2010.10006

[B59] Suzuki T. Hara H. (2011). Role of flavonoids in gut tight junction regulation. J. Nutr. Biochem. 22, 401–408. doi: 10.1016/j.jnutbio.2010.08.001 21167699

[B60] Sveier H. Kvamme B. O. Raae A. J. (2008). Growth and protein utilization in Atlantic salmon (*Salmo salar* L.) given a protease inhibitor in the diet. Aquacult. Nutr. 7, 255–264. doi: 10.1046/j.1365-2095.2001.00183.x37945311

[B61] Tran H. Q. Doan H. V. Stejskal V. (2022). Environmental consequences of using insect meal as an ingredient in aquafeeds: A systematic view. Rev. Aquacult. 14, 237–251. doi: 10.1111/raq.1259540046247

[B62] Turnbaugh P. J. Hamady M. Yatsunenko T. Cantarel B. L. Duncan A. Ley R. E. . (2009). A core gut microbiome in obese and lean twins. Nature 457, 480–484. doi: 10.1038/nature07540 19043404 PMC2677729

[B63] Wang S. Li X. Zhang M. Wang R. X. Qian Y. X. Hong M. L. . (2021). Effects of fermented noni juice on growth, antioxidant status, immune response, gut microbiota and resistance to *Aeromonas hydrophila* of juvenile yellow catfish *Pelteobagrus fulvidraco* fed high‐lipid diet. Aquacult. Nutr. 27, 1290–1303. doi: 10.1111/anu.13268

[B64] Wang X. Luo H. Zheng Y. Wang D. J. Wang Y. X. Zhang W. N. . (2023a). Effects of poultry by-product meal replacing fish meal on growth performance, feed utilization, gut morphology and microbiota communities in juvenile large yellow croaker (*Larimichthys crocea*). Aquacult. Rep. 30, 101547. doi: 10.1016/j.aqrep.2023.10154742574925

[B65] Wang W. Zhang J. Shao R. Liang S. F. Zhang H. Xu W. Q. . (2023b). Sodium acetate alleviates adverse effects caused by the diet with high proportion of soybean meal in turbot (*Scophthalmus maximus* L.). Aquaculture 566, 739163. doi: 10.1016/j.aquaculture.2022.73916342574925

[B66] Wang X. Zhang D. M. Zhang X. P. Lei X. Y. Jiang D. Lin L. L. . (2025). Modulation of growth, gut-liver axis, and antioxidant defense by Bacillus tequilensis (GCB-3) in grass carp (*Ctenopharyngodon idella*) under high-carbohydrate diet: Insights into health enhancement and oxidative stress mitigation. Anim. Feed Sci. Technol. 330, 116530. doi: 10.1016/j.anifeedsci.2025.11653042574925

[B67] Wei Y. Liu X. Huang J. Chen H. He Y. Chen Y. . (2025). Effect of dietary quercetin supplementation on high-starch diet-induced growth impairment, metabolic dysregulation, and oxidative stress in largemouth bass (*Micropterus salmoides*). Aquacult. Rep. 41, 102704. doi: 10.1016/j.aqrep.2025.10270442574925

[B68] Wen H. L. Feng L. Jiang W. D. Liu Y. Jiang J. Li S. H. . (2014). Dietary tryptophan modulates intestinal immune response, barrier function, antioxidant status and gene expression of tor and nrf2 in young grass carp (*ctenopharyngodon idella*). Fish. Shellfish Immunol. 40, 275–287. doi: 10.1016/j.fsi.2014.07.004 25047359

[B69] Willett W. Rockstrom J. Loken B. Springmann M. Lang T. Vermeulen S. . (2019). Food in the anthropocene: the eat-lancet commission on healthy diets from sustainable food systems. Lancet 393, 447–492. doi: 10.0000/lancet.com/PIIS0140-6736(18)31788-4 30660336

[B70] Xie S. W. Yin P. Tian L. X. Yu Y. Y. Liu Y. J. Niu J. . (2020). Dietary supplementation of astaxanthin improved the growth performance, antioxidant ability and immune response of juvenile largemouth bass (*Micropterus salmoides*) fed high-lipid diet. Mar. Drugs 18, 642. doi: 10.3390/md18120642 33333811 PMC7765211

[B71] Xin Y. Li X. Q. Ping K. X. Xiang Y. N. Li M. X. Li X. . (2024). Pesticide avermectin-induced hepatotoxicity and growth inhibition in carp: Ameliorative capacity and potential mechanisms of quercetin as a dietary additive. Aquat. Toxicol. 268, 106859. doi: 10.1016/j.aquatox.2024.106859 38342007

[B72] Xu D. Hu M. J. Wang Y. Q. Cui Y. L. (2019). Antioxidant activities of quercetin and its complexes for medicinal application. Molecules 24, 1123. doi: 10.3390/molecules24061123 30901869 PMC6470739

[B73] Xu F. H. Yang F. Qiu Y. Z. Wang C. S. Zou Q. L. Wang L. Z. . (2024). The alleviative effect of C-phycocyanin peptides against TNBS-induced inflammatory bowel disease in zebrafish via the MAPK/Nrf2 signaling pathways. Fish. Shellfish Immunol. 145, 109351. doi: 10.1016/j.fsi.2023.109351 38171429

[B74] Yang H. Bian Y. H. Huang L. L. Lan Q. Ma L. Z. Li X. Q. . (2022). Effects of dietary quercetin on growth fish meal with fermented soybean meal on the growth performance, gut microbiota, morphology and disease resistance of largemouth bass (*Micropterus salmoides*). Aquacult. Rep. 22, 100954. doi: 10.1016/j.aqrep.2021.10095442574925

[B75] Ye H. Q. Xu M. L. Liu Q. Y. . (2019). Effects of replacing fish meal with soybean meal on growth performance, feed utilization and physiological status of juvenile obscure puffer, *Takifugu obscurus*. Comp. Biochem. Physiol. Part. C. Toxicol. Pharmacol. 216, 75–81. doi: 10.1016/j.cbpc.2018.11.006 30414482

[B76] Zhai S. W. Liu S. L. (2013). Effects of dietary quercetin on growth performance, serum lipids level and body composi tion of tilapia (*Oreochromis niloticus*). Ital. J. Anim. Sci. 12, e85. doi: 10.4081/ijas.2013.e85

[B77] Zhang C. N. Jiang D. X. Wang J. H. Qi Q. (2021). The effects of TPT and dietary quercetin on growth, hepatic oxidative damage and apoptosis in zebrafish. Ecotoxicology Environ. Saf. 224, 112697. doi: 10.1016/j.ecoenv.2021.112697 34450426

[B78] Zhang D. D. Yan Y. N. Tian H. Y. Jiang G. Z. Li X. F. Liu W. B. (2018). Resveratrol supplementation improves lipid and glucose metabolism in high-lipid diet-fed blunt snout bream. Fish Physiol. Biochem. 44, 163–173. doi: 10.1007/s10695-017-0421-9 28891024

[B79] Zhang B. Y. Yuan Z. H. Yu H. X. Yang H. L. Cai G. H. Zhang C. X. . (2025). Functional potential of compound probiotics fermented soybean meal partially replacing fish meal in spotted seabass (*Lateolabrax maculatus*): Insights through growth, immunity, antioxidant capacity and gut health. Aquaculture 598, 742021. doi: 10.1016/j.aquaculture.2024.74202142574925

[B80] Zhao X. T. Gong L. H. Wang C. Liu M. C. Hu N. H. Dai X. Y. . (2021). Quercetin mitigates ethanol-induced hepatic steatosis in zebrafish via P2X7R-mediated PI3K/Keap1/Nrf2 signaling pathway. J. Ethnopharmacol. 268, 113569. doi: 10.1016/j.jep.2020.113569 33186701

[B81] Zhou S. S. Fu Y. X. Yang Q. L. Liu J. H. Li Y. (2025). Mechanism of Quercetin alleviated E. coli K88-induced Liver Injury in Broilers. Poult. Sci. 104, 105828. doi: 10.1016/j.psj.2025.105828 40976095 PMC12489924

[B82] Zhou M. Y. Qiang J. C. Gan J. J. Xu X. H. Li X. Zhang S. . (2023). Quercetin attenuates environmental Avermectin-induced ROS accumulation and alleviates gill damage in carp through activation of the Nrf2 pathway. Comp. Biochem. Physiol. Part. C. Toxicol. Pharmacol. 274, 9. doi: 10.1016/j.cbpc.2023.109744 37704162

[B83] Zhu T. Han M. M. Gu X. K. Liang Y. Zhu C. X. Zhou Z. H. . (2024). The effects of quercetin and taxifolin on gut microbes, digestion enzymes, antioxidant and inflammatory-related gene expression in the Chinese sucker (*Myxocyprinus asiaticus*). Aquacult. Rep. 35, 102011. doi: 10.1016/j.aqrep.2024.10201142574925

