# Supplementary material for: Effects of adding quercetin to soybean meal-based feed on the growth, antioxidant capacity, and gut and liver health of juvenile turbot
Source: Front Physiol. 2026 Oct 28;17:1950062. doi: 10.3389/fphys.2026.1950062 (PMC13751458; doi:10.3389/fphys.2026.1950062)
Supplement: Supplementary file 1 [file SupplementaryFile1.pdf]

Supplementary Material

2.5 Antioxidant Parameters

SOD assay procedure was described as follows. Firstly, 0.05 mL of the test serum was added to the test tube. Subsequently, 1.0 mL of pre-prepared reagent 1 working solution was added to both the test tube and the control tube. Next, 0.05 mL of distilled water was added to the control tube, followed by sequential addition of 0.1 mL each of reagent 2, reagent 3, and pre-prepared reagent 4 working solution to the test tube and control tube. The mixture was thoroughly mixed using a vortex mixer and incubated in a constant-temperature water bath or air bath at 37 °C for 40 min. Finally, 2 mL of the color developer was added to both the test tube and the control tube. An aliquot of 200 μL was transferred from each tube to a 96-well plate, and the absorbance was measured at 550 nm using a microplate reader.

CAT assay procedure was described as follows. Firstly, 100 μL of the test serum was added to the test tube. Subsequently, 1000 μL of reagent 1 was added to both the test tube and the control tube. Then, 100 μL of reagent 2 was supplemented to the two tubes. Timing was initiated immediately after the addition of reagent 2, and the mixtures were mixed thoroughly. After incubation at 37 °C for exactly 1 min, 1000 μL of reagent 3 was immediately added to the test and control tubes, followed by the addition of 100 μL of reagent 4. Finally, 100 μL of distilled water was added to the control tube. An aliquot of 200 μL was transferred from each tube to a 96-well plate, and the absorbance was measured at 405 nm using a microplate reader.

GSH assay procedure was described as follows. Firstly, 100 μL of reagent 1 was added to the blank wells. Subsequently, 100 μL of 20 μmol/LGSH standard solution was added to the standard wells, and 100 μL of the test serum was added to the test wells. Then, 25 μL of reagent 3 was

supplemented to each blank, standard and test well. Finally, 100 μL of reagent 2 was added to each blank, standard and test well. The microplate was shaken gently to homogenize the mixtures, which were allowed to stand for 5 min, and the absorbance of each well was measured at 405 nm using a microplate reader.

T-AOC assay procedure was described as follows. Firstly, 5 μL of distilled water was added to the blank wells. Then, 5 μL of standard solutions with different concentrations was added to the standard wells, and 5 μL of the test serum was added to the assay wells. Subsequently, 180 μL of FRAP working solution was added to the blank wells, standard wells and assay wells. The mixtures were incubated at 37 °C for 3–5 min, and the absorbance value of each well was measured at 593 nm using a microplate reader.

MDA detection procedure was described as follows. Firstly, 25 μL of 10 nmol/mL standard solution was added to the standard tube, 25 μL of anhydrous ethanol was added to the blank tube, and 25 μL of the test serum was added to the test tube. Subsequently, 1 mL of working solution I was sequentially added to the blank tube, standard tube and test tube. The mixtures were thoroughly mixed by vortexing, incubated in an oven at 95 °C for 1 h, and then transferred into cold water for cooling. A small hole was punctured on the lid of each centrifuge tube using a needle. Afterwards, centrifugation was performed at 4,000 rpm for 10 min. An aliquot of 200 μL supernatant was transferred from each tube to a 96-well plate, and the absorbance was measured at 532 nm using a microplate reader.

2.6 Gut Digestibility Indices

TP assay procedure was described as follows. Firstly, 10 μL of distilled water was added to the blank wells. Subsequently, 10 μL of 524 μg/mL standard solution was added to the standard wells, and 10 μL of the test tissue homogenization was added to the test wells. Finally, 250 μL of working

solution was added to each blank, standard and test well. The microplate was shaken gently to mix the mixtures, followed by incubation at 37 °C for 30 min. The absorbance of each well was measured at 562 nm using a microplate reader.

TRY assay procedure was described as follows. Firstly, 0.015–0.05 mL of the test tissue homogenization was added to the test tube. Subsequently, 0.015–0.05 mL of reagent 2 was added to the blank tube. Then, 1.5 mL of substrate solution was added to both the blank tube and the test tube. Timing was initiated when reagent 2 was mixed with the substrate solution, and the mixture was thoroughly homogenized by vortexing before being transferred into a quartz cuvette with a 0.5 cm path length. The absorbance value A was measured at 253 nm using a UV spectrophotometer, and the absorbance value A1 was recorded after 30 s. Afterwards, the reaction solution was transferred from the cuvette to pre-numbered test tubes, which were incubated precisely for 20 min in a water bath maintained at 37 °C. The tubes were taken out, and the absorbance value A2 was measured again after 30 s. $\Delta A = A2 - A1$.

AMS assay procedure was described as follows. Firstly, 5 μL of the test tissue homogenization was added to the test well. Subsequently, 25 μL of reagent 1 was added to both the test well and the blank well. Then, 5 μL of distilled water was added to the blank well, and 205 μL of distilled water was added to the water control well. The microplate was shaken gently to homogenize the mixtures, and the reaction was allowed to proceed for exactly 7.5 min at 37 °C. Finally, 175 μL of iodine reaction solution was added to both the test well and the blank well. The microplate was shaken gently to mix the contents, and the absorbance of each well was measured at 660 nm using a microplate reader.

LPS detection procedure was described as follows. 40 μL of reagent 1, 150 μL of reagent 2, and 10 μL of test tissue homogenization were sequentially added to the test tube. The mixture was

thoroughly mixed, and absorbance A1 was immediately measured at 405 nm and 30 °C. Absorbance A2 was measured after 10 min. ΔA = A2 – A1.

2.11 Serum Physio-biochemical Indices

TG assay procedure was described as follows. Firstly, 2.5 μL of distilled water was added to the blank wells. Then, 2.5 μL of calibrator standard was added to the standard wells, and 2.5 μL of the test serum was added to the sample wells. Subsequently, 250 μL of working solution was added to each blank, standard and sample well. The microplate was shaken for mixing, followed by incubation at 37 °C for 10 min. The absorbance of each well was measured at 500 nm using a microplate reader.

ALB assay procedure was described as follows. Firstly, 2.5 μL of distilled water was added to the blank wells. Then, 2.5 μL of standard solution was added to the standard wells, and 2.5 μL of the test serum was added to the sample wells. Subsequently, 250 μL of pre-prepared reagent 1 working solution was added to each blank, standard and sample well. The microplate was shaken gently to homogenize the mixtures, which were allowed to stand at room temperature for 10 min. The absorbance value of each well was measured at 628 nm or 630 nm using a microplate reader.

ALT assay procedure was described as follows. Firstly, 20 μL of reagent 1 preheated to 37 °C was sequentially added to the test wells and control wells. Subsequently, 5 μL of the test serum was added to the test wells. The microplate was shaken gently for mixing, followed by incubation at 37 °C for 30 min. Then, 20 μL of reagent 2 was sequentially added to the test wells and control wells, and 5 μL of distilled water was added to the control wells. The microplate was shaken gently to homogenize the mixtures, and the reaction was allowed to proceed at 37 °C for 20 min. Finally, 200 μL of stop solution was added to each test well and control well. The microplate was shaken gently to mix the contents, and the mixtures were allowed to stand at room temperature for 15 min. The absorbance of each well was measured at 505 nm using a microplate reader.

AST assay procedure was described as follows. Firstly, 20 μL of reagent 1 preheated to 37 °C was sequentially added to the test wells and control wells. Subsequently, 5 μL of the test serum was added to the test wells. The microplate was shaken gently for mixing, followed by incubation at 37 °C for 30 min. Then, 20 μL of reagent 2 was sequentially added to the test wells and control wells, and 5 μL of distilled water was added to the control wells. The microplate was shaken gently to homogenize the mixtures, and incubation was performed at 37 °C for 20 min. Finally, 200 μL of stop solution was added to each test well and control well. The microplate was shaken gently to mix the contents, and the mixtures were allowed to stand at room temperature for 15 min. The absorbance of each well was measured at 505 nm using a microplate reader.

AKP assay procedure was described as follows. Firstly, 5 μL of double-distilled water was added to the blank well. Subsequently, 5 μL of 0.1 mg/mL phenol standard solution was added to the standard well, and 5 μL of the test serum was added to the test well. Then, 50 μL of buffer and 50 μL of substrate solution were respectively added to the blank well, standard well and test well. The microplate was shaken gently to homogenize the mixtures, followed by incubation in a water bath at 37 °C for 15 min. Finally, 150 μL of color developer was sequentially added to the blank, standard and test wells. The microplate was shaken gently to mix the contents, and the absorbance of each well was measured at 520 nm using a microplate reader.

γ-GT assay procedure was described as follows. Firstly, 15 μL of distilled water was added to the blank wells. Subsequently, 15 μL of standard solution was added to the standard wells, and 15 μL of the test serum was added to the test wells. Then, 200 μL of reagent 1 was sequentially added to the blank wells, standard wells and test wells. The microplate was shaken gently to homogenize the mixtures, followed by incubation at 37 °C for 5 min. Finally, 50 μL of reagent 2 was added to each blank, standard and test well. The microplate was shaken gently for mixing, and incubation was

performed at 37 °C for 1 min. The variations in absorbance of each well were continuously monitored at 415 nm using a microplate reader over 1–3 min to calculate ΔA.
